# A novel role for transcription factor NFE2 in redox regulation and chemotherapy resistance in acute myeloid leukemia

**DOI:** 10.1038/s41375-026-03095-x

**Published:** 2026-08-19

**Authors:** Anne Marie Staehle, Florian Perner, Hans Felix Staehle, Katharina Gellrich, Fiona Preiß, Annika Grafemeyer, Jana Schulze, Jean-Luca Funk, Albert Gründer, Julia Berner, Sander Bekeschus, Christoph Koellerer, Heike L. Pahl

**Affiliations:** 1 Department of Hematology/Oncology, Medical Center - University of Freiburg, 79106 Freiburg, Germany; 2 Hematology, Hemostasis, Oncology, and Stem Cell Transplantation, Hannover Medical School, Hannover, Germany; 3 Faculty of Biology - University of Freiburg, 79106 Freiburg, Germany; 4 Leibniz Institute for Plasma Science and Technology (INP), ZIK Plasmatis, Greifswald, Germany; 5 Rostock University Medical Center, Clinic and Policlinic for Dermatology, Venerology and Allergology, Rostock, Germany

**Keywords:** Acute myeloid leukaemia, Cancer therapeutic resistance

## Abstract

Elevated activity of transcription factor NFE2 is sufficient to cause leukemic transformation in absence of a classical leukemic driver. However, the molecular mechanism promoting NFE2-driven leukemogenesis is not known. Here we report a previously unrecognized role for NFE2 as a key regulator of the oxidative stress response in leukemic cells. Through a comprehensive analysis of NFE2 genomic occupancy and its effect on chromatin accessibility and transcription, we demonstrate that NFE2 regulates glutathione homeostasis as well as the expression of central detoxifying enzymes. NFE2 constituted one of the highest scoring gene dependencies in MLL-AF9-transformed primary leukemic cells under redox stress, substantially more significant than the universally recognized redox regulator NRF2. Consequently, NFE2 knockdown sensitized leukemic cells to GSH depletion, ferroptosis induction, as well as to cytarabine treatment. Deriving an NFE2 RedOx score, we demonstrate that increased NFE2 activity constitutes an independent predictor of inferior outcome in AML patients at diagnosis.

## Introduction

Expression of the transcription factor NFE2 is increased in patients with myeloid neoplasms [1, 2]. In addition, a subset of patients with MPN and AML carry NFE2 mutations that augment transcription factor activity [3, 4]. Increased NFE2 levels are leukemogenic in several murine models, as they are sufficient to cause an acute leukemia in absence of a classical AML driver [5]. Moreover, Marcault et al. have recently shown that MPN patients carrying an NFE2 mutation incur a 10-fold increased risk of leukemic transformation [6], which translates to a significantly reduced overall survival.

To date, NFE2 is mainly recognized as an erythrocytic and megakaryocytic transcription factor and potential functions outside these lineages have been poorly characterized. In particular, the molecular mechanism by which increased NFE2 activity causes leukemic transformation is not known. We therefore performed a comprehensive analysis of NFE2 genomic occupancy and its effect on chromatin accessibility and transcription.

NFE2 is a member of the Cap’n’collar (CnC) family of transcription factors which also includes NRF2 (NFE2-related factor 2). While NFE2 is primarily expressed in hematopoietic cells, NRF2 is ubiquitously expressed and is well-characterized as a central regulator of the oxidative stress response [7]. Increased resistance of AML cells to oxidative stress plays an important part in disease initiation and persistence and this characteristic has previously been attributed to increased NRF2 activity [8]. Here we report for the first time that NFE2 constitutes a key regulator of the oxidative stress response in leukemic cells.

## Materials and methods

Detailed experimental methods are described in the Supplemental Methods, available on the *Leukemia* website.

### Cell lines

HEL and HL-60 cells were obtained from DSMZ. CB3 cells were a kind gift of V. Blank and E. Bresnick.

### Cytarabine, daunorubicine and parthenolide treatments

Cells were treated with cytarabine, daunorubicine or parthenolide 48 h after transduction for 48 h. Viability was assessed using CellTiter-Glo®.

### Ferroptosis assay

Cells were treated with the ferroptosis inducer ML-210 for 24 h, with or without simultaneous inhibition with 10 mM ferrostatin. Cell death was assessed using AnnexinV-PI flow cytometry.

### Genome-wide CRISPR-Cas9 screening

Murine Lin-/Kit + /Sca1+ cells were transformed using MLL-AF9 (KMT2A::MLLT3) [9]. A genome wide library (*n* = 92,919 sgRNAs) was transduced and cells were treated with H_2_O_2_ or PBS for 10 days in triplicates.

### Chromatin Immunoprecipitation (ChIP-seq)

ChIP-seq was performed as previously described [4, 10].

### Assay for Transposase-Accessible Chromatin (ATAC-seq)

We performed Omni-ATAC as previously described [11].

### Statistical analysis

Two-tailed, unpaired Student’s t-tests were used to determine whether a significant (*p* < 0.05) difference existed between two groups, unless otherwise stated.

### Publicly available AML datasets used

BEAT2 [12], TARGET AML [13], TCGA LAML [14], Verhaak et al. [15], Jayavelu et al*.* [16].

## Results

### The effect of NFE2 overexpression on the transcriptome

**Fig. 1:**
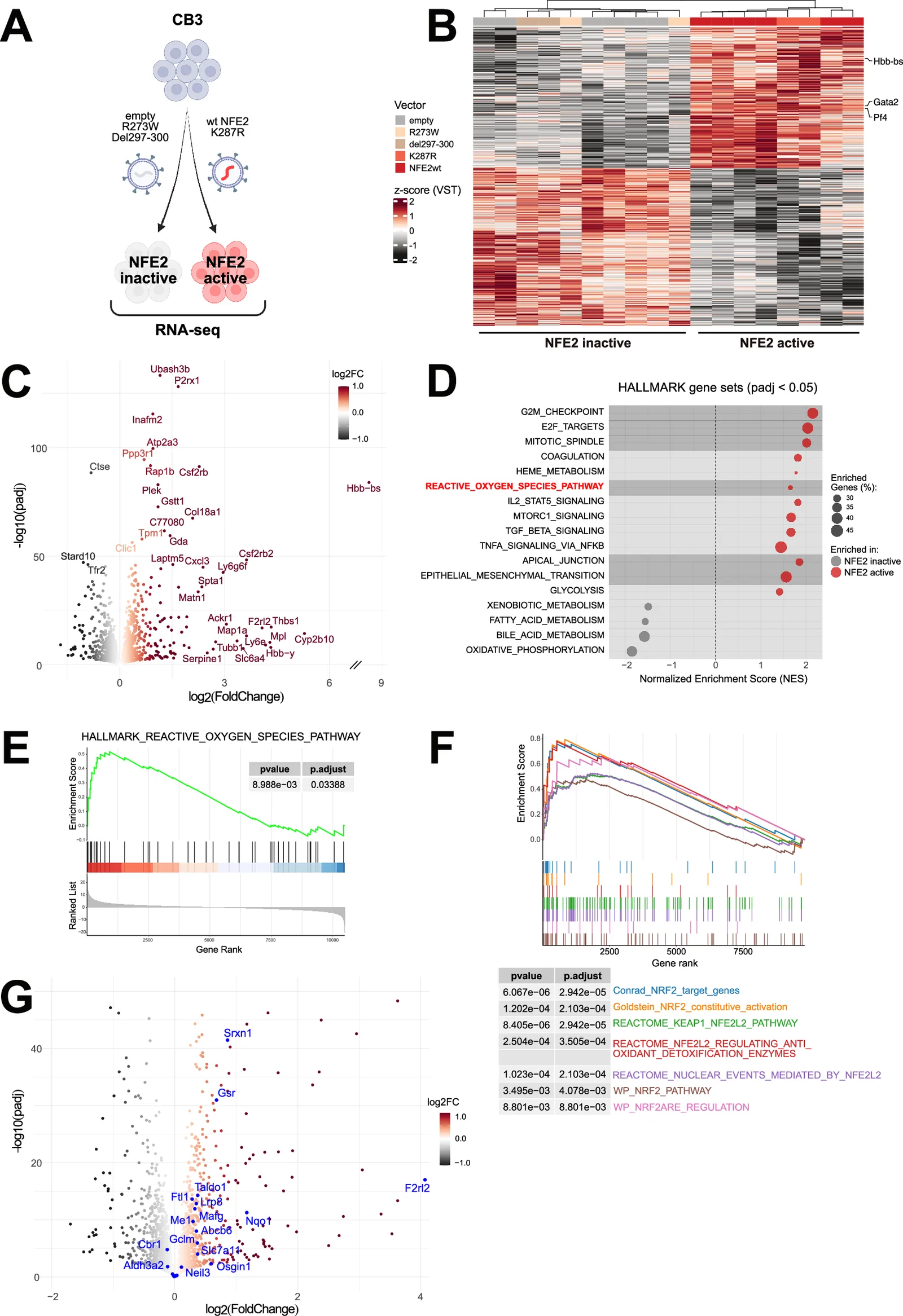
Overexpression of NFE2 in leukemic cells leads to upregulation of canonical NRF2 target genes involved in oxidative-stress responses. **A** Experimental design of RNA-seq experiments reported in panels B-G. CB3 cells were lentivirally transduced with control vectors ( = “NFE2 inactive“) or vectors conferring NFE2 overactivity ( = “NFE2 active“). “NFE2 inactive“: empty (*n* = 6 biological replicates), NFE2-R273W (*n* = 2 biological replicates), NFE2-Del297-300 (*n* = 2 biological replicates). “NFE2 active“: wt NFE2 (*n* = 6 biological replicates), NFE2-K287R (*n* = 2 biological replicates). RNA was isolated after 72 h. Created in BioRender. Pahl, H. (2026) https://BioRender.com/gx655wv**B** Heatmap of z-scaled, vst-transformed counts of differentially expressed genes between “NFE2 active“ and “NFE2 inactive“ conditions (p_adj_ < 0.05). Previously described NFE2 target genes are highlighted. **C** Volcano plot depicting gene expression changes between “NFE2 active“ and “NFE2 inactive“ conditions. **D** Gene Set Enrichment Analysis (GSEA) of mSigDB Hallmark gene sets. Cut off for depicted Gene Sets: *q* < 0.05. **E** GSEA plot for the HALLMARK “Reactive Oxygen Species Pathway“ gene set. **F** Combined GSEA plots of NRF2-related gene sets from curated databases (Reactome, WikiPathways) as well as two previously reported NRF2 signature gene sets [19, 20]. **G** Genes from the Conrad et al. NRF2 gene set highlighted in the volcano plot from panel C (axes adjusted). **A**–**G** Significance of differential gene expression was assessed using DESeq2 (Wald test).

In order to assess the downstream effects of NFE2 activity, we used CB3 cells, which are naturally devoid of NFE2 activity due to viral integration [17], but are primed to express NFE2 target genes upon reintroduction of the transcription factor. CB3 cells were lentivirally transduced with either an empty control vector, or lentiviruses expressing one of the following NFE2 cDNA constructs (Fig. 1A): NFE2 wild-type (wt), NFE2-R273W, NFE2-Del297-300 or NFE2-K287R. While wt NFE2 and NFE2-K287R confer NFE2 activity, the NFE2-R273W and NFE2-Del297-300 mutant are inactive when expressed on their own (Fig. 1B and Supplementary Fig. 1A, B) [4]. The latter therefore constitute additional controls for true NFE2 transcriptional activity.

Following RNA-seq analysis, we compared gene expression in cells with active NFE2 to cells devoid of NFE2 activity (Fig. 1B, Supplementary Table 1). At a significance level of p_adj_ < 0.05, a total of 2841 genes were differentially expressed between “NFE2 active” and “NFE2 inactive” cells. These included previously described NFE2 target genes, for example hemoglobin beta, Gata2 and platelet factor 4, consistent with NFE2’s recognized role in erythroid and megakaryocytic maturation (Fig. 1B, C). However, it was unexpected that upon gene set enrichment analysis (GSEA) NFE2 up-regulated genes were enriched in the hallmark Reactive Oxygen Species gene set (Fig. 1D, E). A role for NFE2 in promoting antioxidant and stress-response pathways in leukemic cells has not been previously described.

### NFE2 induces expression of classical NRF2 target genes

A transcription factor closely related to NFE2, the NFE2-related factor 2 (NRF2), regulates the expression of numerous antioxidant and detoxification genes. NRF2 binds the “antioxidant response element” ARE, which is similar to the sequence bound by NFE2-Maf heterodimers. CB3 cells show low NRF2 protein levels, which are not altered by NFE2 overexpression (Supplementary Fig, 1C).

Campbell et al. have previously suggested that NFE2 and NRF2 may bind similar DNA sites [18]. We therefore identified two published NRF2 gene signatures (Conrad et al. [19], Goldstein et al. [20]) as well as five gene sets containing NRF2 target genes in the REACTOME and WikiPathways collections and show that all seven gene sets are significantly enriched in genes upregulated by NFE2 (Fig. 1F). This strongly suggests that classical NRF2 target genes are also regulated by NFE2. In support, we show that many classical NRF2 target genes identified by Conrad et al. [19] are strongly and significantly upregulated upon NFE2 expression in CB3 cells (Fig. 1G and Supplementary Fig. 1D). We therefore hypothesize a novel role for NFE2 in regulating antioxidant response by co-regulating genes encoding detoxifying proteins.

### NFE2 directly binds and transactivates antioxidant response genes

**Fig. 2:**
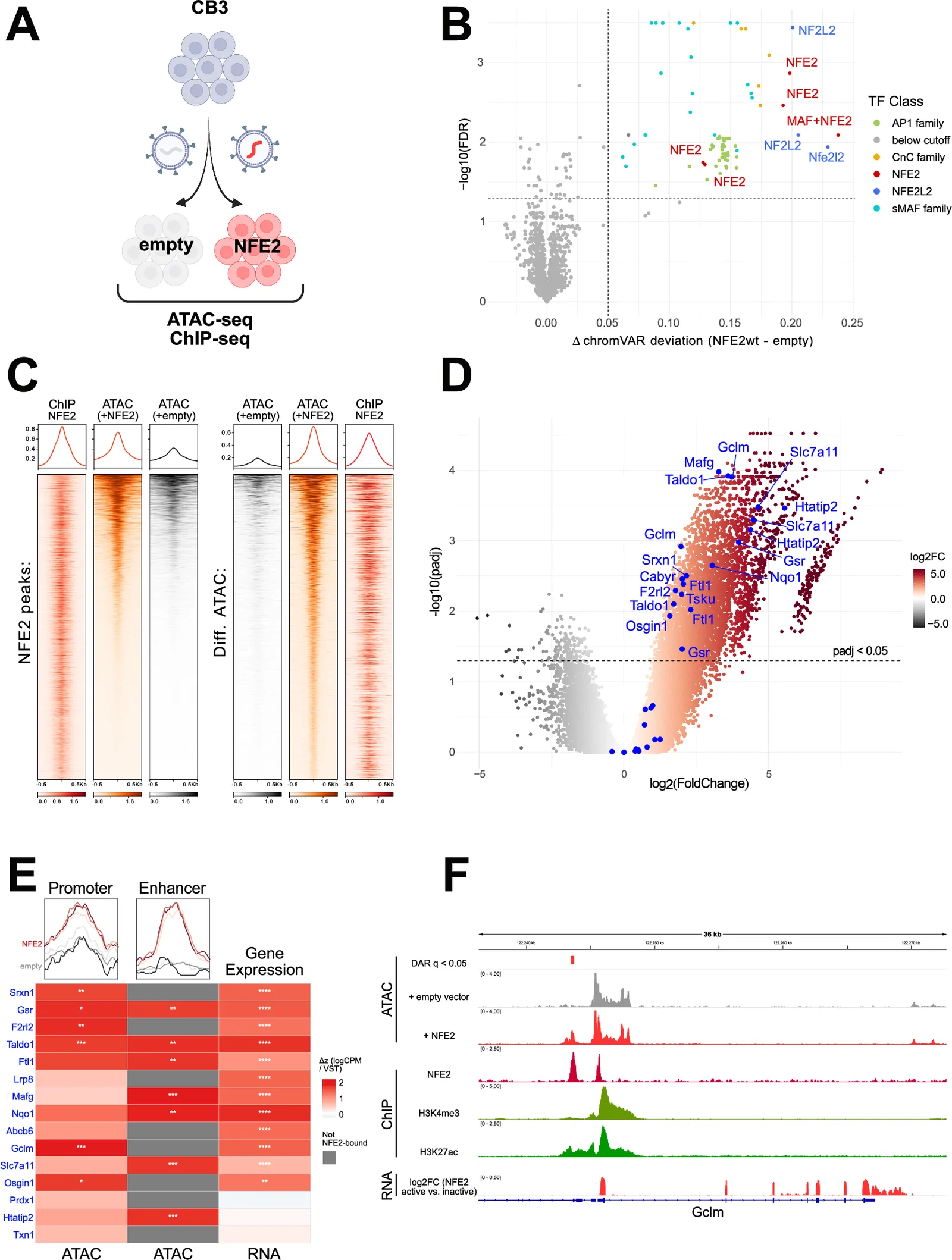
NFE2 binds the cis-regulatory elements of canonical NRF2 target genes and leads to increased chromatin accessibility. **A** Experimental design of ATAC- and ChIP-seq experiments reported in panels B-F. CB3 cells were lentivirally transduced with an empty control vector or a vector overexpressing wt NFE2 and used for ATAC-seq (*n* = 3 biological replicates per condition) and ChIP-seq (*n* = 1). Created in BioRender. Pahl, H. (2026) https://BioRender.com/fha8k6v. **B** Differential transcription factor motif accessibility between ATAC-seq from control cells and cells overexpressing wt NFE2 calculated with chromVAR. Dashed lines indicate cutoffs at False Discovery Rate (FDR) < 0.05 and delta(deviation) > 0.05. Statistical testing was performed via Welch’s unequal-variance *t*-test, followed by Benjamini-Hochberg (BH) correction. **C** Heatmaps of ATAC and NFE2 ChIP coverage signal at high-confidence NFE2 ChIP-seq peaks (left: *n* = 8500 regions) and at loci with increased accessibility between control and NFE2-overexpressing cells (right: *n* = 4324 regions). Coverage scores were calculated in counts per million (CPM). The reported coordinates were generated using significance thresholds of: FDR < 0.05 in the case of ATAC-Seq (quasi-likelihood F-test, BH-corrected) and *p* < 10^-5^ in the case of ChIP-seq (MACS2). **D** Volcano plot of differential chromatin accessibility changes between control and NFE2-expressing CB3 cells. Cis-regulatory elements (CREs) were assigned to the nearest gene and NFE2-bound CREs corresponding to NRF2 targets from the Conrad et al. data set are labeled in blue. **E** Heatmap of ATAC- and RNA-seq signals at the loci of NRF2 target genes with NFE2-bound promoters. ATAC peaks within 2 kb of the transcription start site (TSS) were labeled “Promoter”, intragenic peaks and peaks up to 5 kb upstream of TSS were labeled “Enhancers”. For CREs overlapping NFE2 ChIP peaks z-scaled log (CPM) are shown, for RNA expression z-scaled VST-transformed counts are shown. **F** Exemplary ATAC-, ChIP- and RNA-seq signal tracks at the *Gclm* locus. ChIP and ATAC coverage were normalized to CPM, the ATAC track corresponds to the mean of *n* = 3 replicates and the RNA-seq track represents the log2 fold changes between NFE2-transduced and control cells. H3K4me3 and H3K27ac ChIP-seq (*n* = 1 each) was performed in NFE2-transduced CB3 cells. Red bars indicate differentially accessible regions at FDR < 0.05. **A**–**F** *p_adj_ < 0.05, **p_adj_ < 0.01, ***p_adj_ < 0.001, ****p_adj_ < 0.0001. Quasi-likelihood F-testing followed by BH-correction was used to assess significance of differential chromatin accessibility, differential gene expression was tested by Wald-test.

In order to determine whether NFE2 indeed binds to and affects chromatin accessibility at loci encoding classical NRF2 target genes, we conducted ChIP-seq and ATAC-seq analyses in CB3 cells transduced with either an empty control vector or a vector expressing the NFE2 cDNA (Fig. 2A). Upon expression of NFE2, chromatin accessibility was significantly increased at 4329 sites, suggesting that the presence of NFE2 alone is sufficient to alter chromatin accessibility (Supplementary Table 2). Further supporting the transcription factor’s ability to confer changes in chromatin configuration, we show that in the presence of NFE2, the DNA binding motifs of both NFE2 and NRF2 become highly accessible (Fig. 2B and Supplementary Fig. 2). In addition, DNA binding motifs of the other Cap’n’collar (CnC) transcription factors, NRF1 and NRF3, as well as the activator protein 1 (AP1) motif, and the Maf recognition element (MARE) become accessible in the presence of NFE2 (Fig. 2B). These data clearly demonstrate that introduction of NFE2 into CB3 cells is sufficient to confer an open chromatin conformation at DNA sites harboring the recognition sequences for NRF2, other CnC transcription factors, as well as Maf and AP1 family transcription factors.

Next, we identified NFE2 DNA binding sites, calling 8500 high-confidence NFE2 ChIP-seq peaks in NFE2 expressing CB3 cells, and integrated this data with chromatin accessibility data from the ATAC-seq. The majority of NFE2 bound sites showed an increase in chromatin accessibility (Fig. 2C, left). Vice versa, the majority of differentially accessible loci are physically bound by NFE2 (Fig. 2C, right). Importantly, the majority of cis-regulatory elements (CREs) associated with classical NRF2 targets show increased chromatin accessibility in the presence of NFE2 (Fig. 2D). Consistent with this, nearly all NRF2 targets whose promoters are bound by NFE2, show increased accessibility at their promoter and/or enhancer regions in the presence of NFE2 (Fig. 2E**)**. Figure 2F shows an example of NFE2 DNA binding, consecutive increased DNA accessibility and increased RNA transcription at the *Gclm* locus. *Gclm*, which encodes the modifier subunit of glutamate-cysteine ligase (GCL), the rate-limiting enzyme in glutathione (GSH) synthesis, represents a canonical NRF2 target [19]. These data strengthen our postulated novel role for NFE2 in directly transcriptionally regulating antioxidant and stress-response pathways.

### NFE2 and NRF2 share the majority of their binding sites

To directly assess whether NFE2 and NRF2 co-localize at specific genomic sites, we conducted in silico analysis on NFE2 and NRF2 ChIP-seq data from K562 cells. We demonstrate that the two transcription factors co-localize at a large proportion of their respective binding sites (*p* < 10^-10^, Supplementary Fig. 3A). The *Gclm* locus, the classical NRF2 target gene identified as a novel NFE2 target above (Fig. 2F), is shown as an example (Supplementary Fig. 3B). Mining NFE2 and NRF2 ChIP-seq data from five different hematopoietic and non-hematopoietic human cell lines to conduct transcription factor motif analysis, we show that NFE2 and NRF2 indeed share the same core binding motif (Supplementary Fig. 3C). While this had previously been postulated, large scale data analysis has so far been scarce. Moreover, the available data only investigated each transcription factor in isolation, the effect of simultaneous NFE2 and NRF2 activity has not been determined so far.

### NFE2 and NRF2 cooperate in modifying chromatin accessibility

**Fig. 3:**
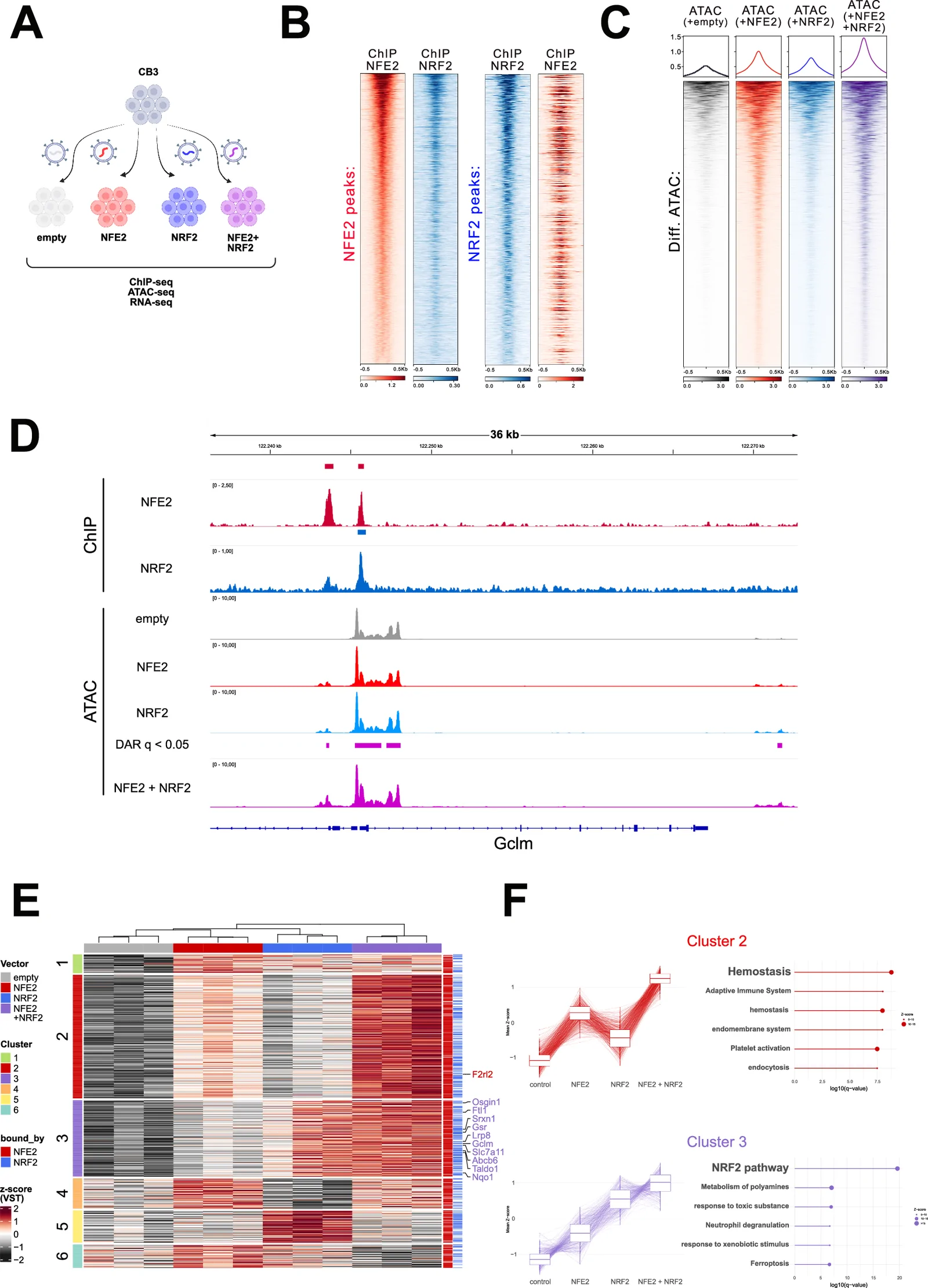
NFE2 and NRF2 share the majority of their binding sites, jointly affecting chromatin accessibility and co-regulating functionally distinct sets of genes. **A** Experimental design of experiments reported in panels B-F. CB3 cells were lentivirally transduced with different combinations of empty, NFE2 and NRF2 vectors and used for ChIP-seq (*n* = 1), ATAC-seq (*n* = 3 biological replicates) and RNA-seq (*n* = 3 biological replicates). In the case of NRF2 we designed a custom construct that is resistant to KEAP1-mediated proteasomal degradation and C-terminally fused to an AM-tag to allow specific immunoprecipitation. Created in BioRender. Pahl, H. (2026) https://BioRender.com/pqii1yy**B** Heatmaps of NFE2 and NRF2 ChIP-seq coverage signal (counts per million, CPM) in 1 kb windows centered around NFE2 (left: *n* = 4846 regions) and NRF2 peaks (right: *n* = 725 regions) at *p* < 10^-5^ that overlapped cis-regulatory elements (CREs). **C** Heatmaps of ATAC signal (CPM) in 1 kb windows around a union peakset of DARs (p_adj_ < 0.05) between empty controls and either NFE2- or NRF2-overexpressing cells (*n* = 4015 regions). **D** Representative ChIP- and ATAC-seq tracks are shown at the *Gclm* locus. Coverage scores were calculated as CPM. ChIP-seq peaks were called at *p* < 10^-5^. Purple bars display differentially accessible regions (DARs) between the NRF2-overexpressing condition and the condition overexpressing both vectors at p_adj_ < 0.05. **E** Heatmap of differentially expressed NFE2 and NRF2 target genes across conditions. Differentially expressed genes at p_adj_ < 0.05 between NFE2- or NRF2-overexpressing conditions compared to control conditions were filtered for genes associated with NFE2 or NRF2 ChIP-seq peaks (*p* < 10^-5^). Normalized RNA-seq counts were vst-transformed and z-scaled. Genes were grouped by the consensus of 1,000 bootstrapped k-means clusterings, samples were organized by hierarchical clustering using euclidean distance. **F** Boxplot of vst-transformed, z-scaled counts for clusters 2 and 3 defined in (**E**) across conditions (left). Dotplots of gene set enrichment analysis (right) performed on the members of each cluster using Metascape. The six highest-ranking gene sets by significance are displayed. **A**–**F** Quasi-likelihood F-testing followed by Benjamini-Hochberg correction was used to assess significance of differential chromatin accessibility, differential gene expression was tested by Wald-test.

We therefore sought to determine the effect of concomitant NFE2 and NRF2 activity on the transcriptome. For this, we extended our experimental approach (Fig. 3A). CB3 cells were transduced with vectors expressing either NFE2 or a degradation resistant NRF2 in isolation or co-transduced to express both transcription factors simultaneously (Supplementary Fig. 4A-C). These three conditions were compared to cells transduced with an empty control vector using ChIP-, ATAC- and RNA-seq experiments (Supplementary Fig. 5A-C, Supplementary Table 3).

We assessed transcription factor binding in CB3 by examining the called NFE2 peaks for NRF2 binding and, vice versa the called NRF2 peaks for NFE2 binding. Figure 3B shows that NFE2 and NRF2 co-localize at the majority of bound sites. Analysis of *n* = 4015 regions with differentially increased ATAC-seq signals upon overexpression of either NFE2 or NRF2 revealed that most sites showed greater accessibility when both transcription factors were present, indicating frequent cooperative activity of NFE2 and NRF2 in promoting transcription (Fig. 3C, Supplementary Fig. 5D-E). This is noteworthy as the opposite, an antagonistic effect of these transcription factors on the transcription of cytoprotective genes, was previously postulated [21]. Investigation of the NFE2 interactome did not reveal protein-protein interaction between the two transcription factors (Supplementary Tables 4 and 5, Supplementary Fig. 6). The *Gclm* locus is again shown as an example of NFE2 and NRF2 co-localization and its effect on chromatin accessibility of regulatory elements (Fig. 3D). Notably, co-expression of both transcription factors increased ATAC-seq accessibility at the *Gclm* locus beyond that observed with either factor alone, indicating cooperative establishment of open chromatin at this target gene (Fig. 3D, lower tracks).

### NFE2 and NRF2 target genes form distinct expression clusters

It was therefore essential to determine the effect of NFE2 and NRF2 co-expression on the transcription of target genes. Genes differentially expressed between control and NFE2- or NRF2-overexpressing conditions, and also bound by either of the transcription factors in ChIP-seq, were subjected to unsupervised clustering. Six distinct clusters can be distinguished (Fig. 3E and F, Supplementary Fig. 5F). Four clusters (1,4-6) are small and show only weak enrichments in innate immunity and stress responses. Clusters 2 and 3 warrant special attention, as they challenge previously published models and depict novel roles for NFE2 in regulating cellular homeostasis (Fig. 3F). Cluster 2, depicted in red (Fig. 3F, top panels), contains classical NFE2 target genes, for example hemoglobin beta and platelet factor 4, and is enriched for genes involved in platelet activation. Expression of these genes is slightly induced in the presence of NRF2 alone, but we show here for the first time that NRF2 cooperates with NFE2 to increase transcription of these canonical NFE2 targets (Fig. 3F, top panels). Cluster 3, depicted in violet, contains a large number of stress-response genes, previously defined as classical NRF2 targets, for example *Gclm* and *Nqo1*. Notably, transcription of these genes is also upregulated in the presence of NFE2 alone, and, contrary to previously published data [21], transcription is increased above the level of that mediated by NRF2 alone when both transcription factors are present (Fig. 3F, bottom panels and Supplementary Fig. 7). Hence, contrary to the previously published model, NFE2 cooperates with NRF2 to promote expression of redox-regulatory genes. Multiomics analysis therefore suggests a previously unrecognized role for NFE2 in regulating cellular stress-response.

### NFE2 constitutes a key regulator of glutathione homeostasis

**Fig. 4:**
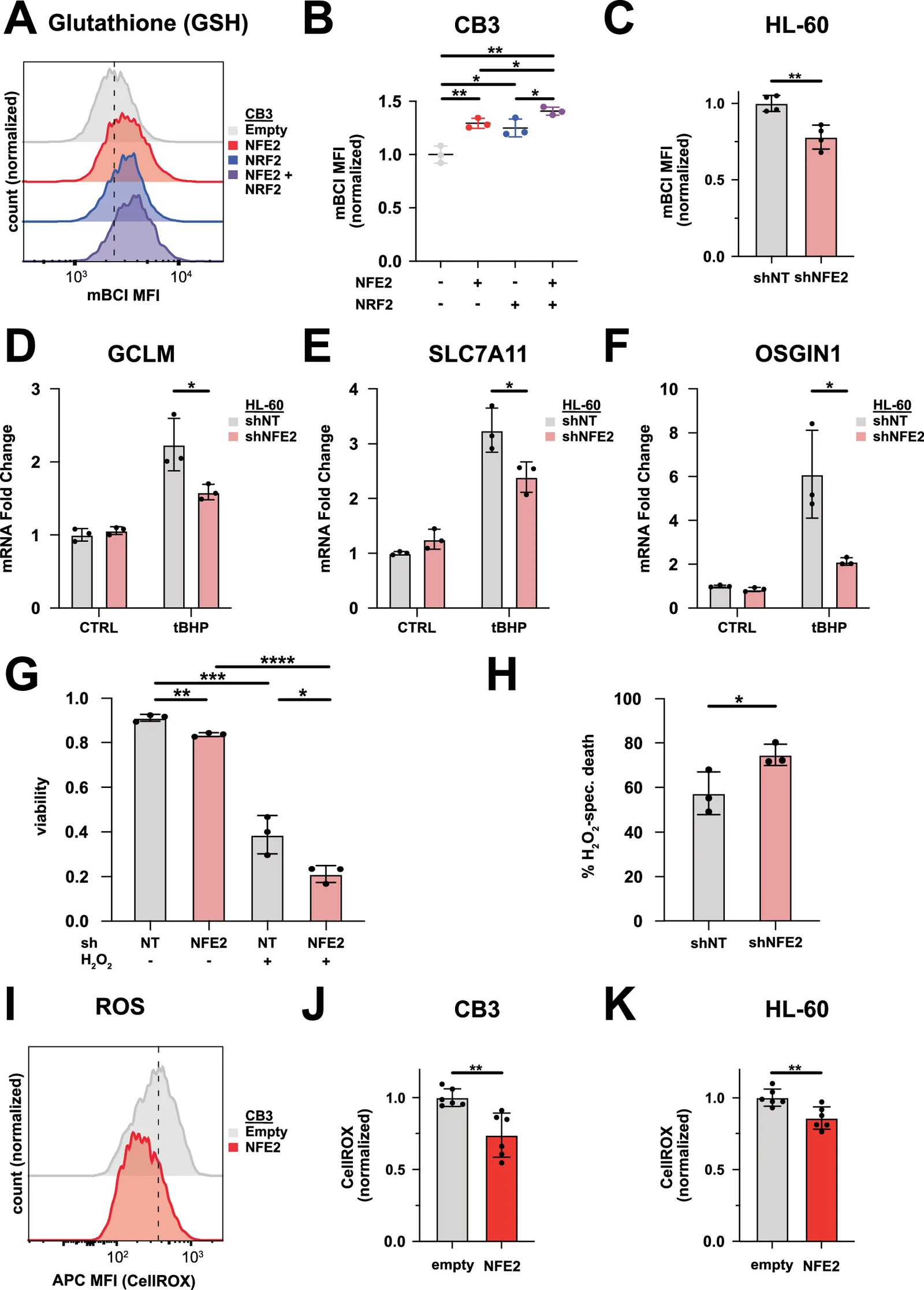
NFE2 regulates cellular glutathione and ROS levels and mediates resistance to reactive oxygen species. **A**, **B** CB3 cells were lentivirally transduced with different combinations of empty, NFE2 and NRF2 vectors. Reduced glutathione levels were measured after 48 h by flow cytometry using monochlorobimane (mBCI); *n* = 3 biological replicates per condition. Gating strategy depicted in Supplementary Fig. 9A. **A** Representative flow cytometry histograms of mBCI fluorescence. **B** Dot plots showing mean fluorescence intensity (MFI) values normalized to the mean MFI of the empty vector control. **C**–**H** HL-60 cells were transduced with lentiviral vectors expressing a non-targeting shRNA (shNT) or an shRNA targeting NFE2 (shNFE2). **C** Reduced glutathione levels measured by mBCI-based flow cytometry, *n* = 4 biological replicates per condition. Gating strategy depicted in Supplementary Fig. 9E. **D**–**F** Expression of GCLM(**D**), SLC7A11(**E**) and OSGIN1(**F**) measured by RTqPCR after a 4-hour treatment with vehicle (CTRL) or tert-butyl hydroperoxide (tBHP), *n* = 3 biological replicates per condition. Fold Changes were calculated with respect to the shNT CTRL. **G** Cell viability following 48 h treatment with 50 μM H₂O₂ or vehicle, assessed by Annexin V/PI staining. Viability calculated as the percentage of Annexin V⁻/PI⁻ cells, *n* = 3 biological replicates per condition. Gating strategy depicted in Supplementary Fig. 9F. **H** H₂O₂-specific cell death calculated relative to vehicle-treated controls (see Methods for details), *n* = 3 biological replicates per condition. **I**–**K** CB3 (**I**, **J**) and HL-60 (**K**) cells were lentivirally transduced with an empty vector or a vector expressing NFE2. Reactive oxygen species (ROS) were measured after 48 or 72 h by flow cytometry using CellROX Deep Red, *n* = 6 biological replicates per condition. Gating strategies are depicted in Supplementary Fig. 10. **I** Representative flow cytometry histograms of APC fluorescence (CellROX). **J–K** MFI values, normalized to the mean MFI of the empty vector control. **B**–**H**, **J**, **K** Mean depicted with standard deviation (SD). Statistical analysis by two-sided unpaired t-test. *p < 0.05, **p < 0.01, ***p < 0.001, ****p < 0.0001.

As several of the novel NFE2 targets identified constitute enzymes involved in glutathione homeostasis, including the rate limiting GCLM subunit (Supplementary Fig. 8), we hypothesize a novel role of NFE2 in regulating glutathione homeostasis. We therefore assessed glutathione levels in control transduced CB3 cells as well as in CB3 cells expressing NFE2, NRF2 or both transcription factors. Parallel to the mRNA expression of several glutathione synthetic enzymes, GSH levels increased upon expression of either NFE2 or NRF2 alone, both to equal amounts, but rose even more in the presence of both transcription factors (Fig. 4A, B, Supplementary Fig. 9A). Conversely, silencing NFE2 via RNAi in HL-60 cells lowered glutathione levels significantly, demonstrating that NFE2 activity is required for maintaining maximal levels of the antioxidant metabolite (Fig. 4C, Supplementary Fig. 9B–E). NFE2 depletion also diminished redox-stress induced expression of the antioxidant genes GCLM, SLC7A11 and OSGIN1, suggesting that leukemic cells require endogenous NFE2 for maximum redox stress detoxification (Fig. 4D–F**)**. Fitting with this model, HL-60 became significantly less resistant to H_2_O_2_ treatment following NFE2 depletion, demonstrating the functional consequence of diminished NFE2 levels on resistance to oxidative stress (Fig. 4G, H, Supplementary Fig. 9F). Moreover, overexpression of NFE2 caused a decrease of endogenous ROS levels in both HL-60 and CB3 cells (Fig. 4I–K, Supplementary Fig. 10**)**.

### NFE2 constitutes a top redox-specific gene dependency in MLL-AF9-transformed primary cells

**Fig. 5:**
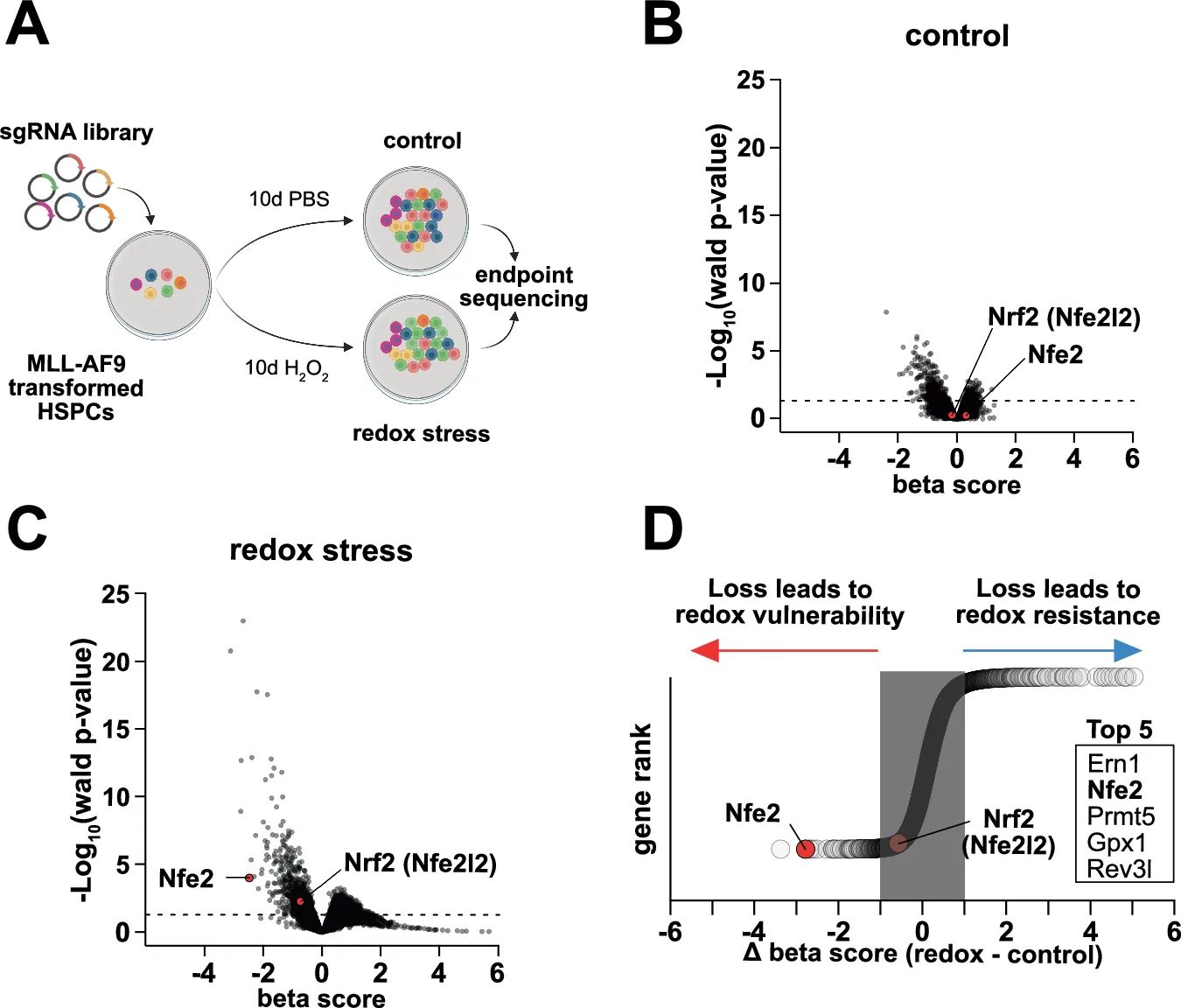
NFE2 constitutes a central redox-specific dependency of primary leukemic cells. **A** Experimental setup used for panels A-D. Primary murine hematopoietic stem and progenitor cells (HSPCs) were lentivirally transduced with an MLL-AF9 (KMT2A::MLLT3) construct. The cells were then transduced with the M1 “Liu Mouse CRISPR Knockout Library” for expression of 92,919 sgRNA constructs along with the Cas9 protein. Following transduction, the cells were cyclically treated with H_2_O_2_ (1x/d 100 µM, “redox stress” condition) or PBS (1x/d, “control” condition) for 10 d and then harvested for endpoint sequencing, *n* = 3 biological replicates per condition. Created in BioRender. Pahl, H. (2026) https://BioRender.com/rnibtzn. **B**, **C** Volcano plot depicting beta-scores and wald p-values of all genes targeted by the sgRNA library in the control (**B**) and the redox stress (**C**) condition. *Nfe2* and *Nrf2* (*Nfe2l2)* are marked in red. **D** Genes ranked by ∆ beta score = beta score (redox stress) – beta score (control) in ascending order. Gray area marks genes with a ∆ beta score between -1 and 1. Box contains names of the top 5 ranked genes.

To assess the relative importance of NFE2 activity on resistance to oxidative stress in an unbiased fashion, we transduced MLL-AF9 transformed murine bone marrow stem and progenitor cells with a genome-wide lentiviral CRISPR-sgRNA-library. Cells were cultured for ten days with the daily addition of either PBS (control) or 100 μM H_2_O_2_, to produce redox stress. Genomic DNA of surviving cells was sequenced to reveal differences between sgRNA abundance in the two conditions (Fig. 5A, Supplementary Table 6). In control cultures, loss of NFE2 or NRF2 by sgRNA insertion did not affect cell proliferation or viability (Fig. 5B). In contrast, cells devoid of either transcription factor were significantly depleted following ten days of culture with daily addition of H_2_O_2_ (Fig. 5C), strongly suggesting that the absence of either NFE2 or NRF2 renders cells more susceptible to death by oxidative stress. Unexpectedly, the loss of NFE2 rendered murine leukemic cells more vulnerable to redox stress than the loss of NRF2, previously thought of as the most important transcription factor regulating oxidative stress response (Fig. 5C, D).

### NFE2 depletion renders AML cells more susceptible to ferroptosis

**Fig. 6:**
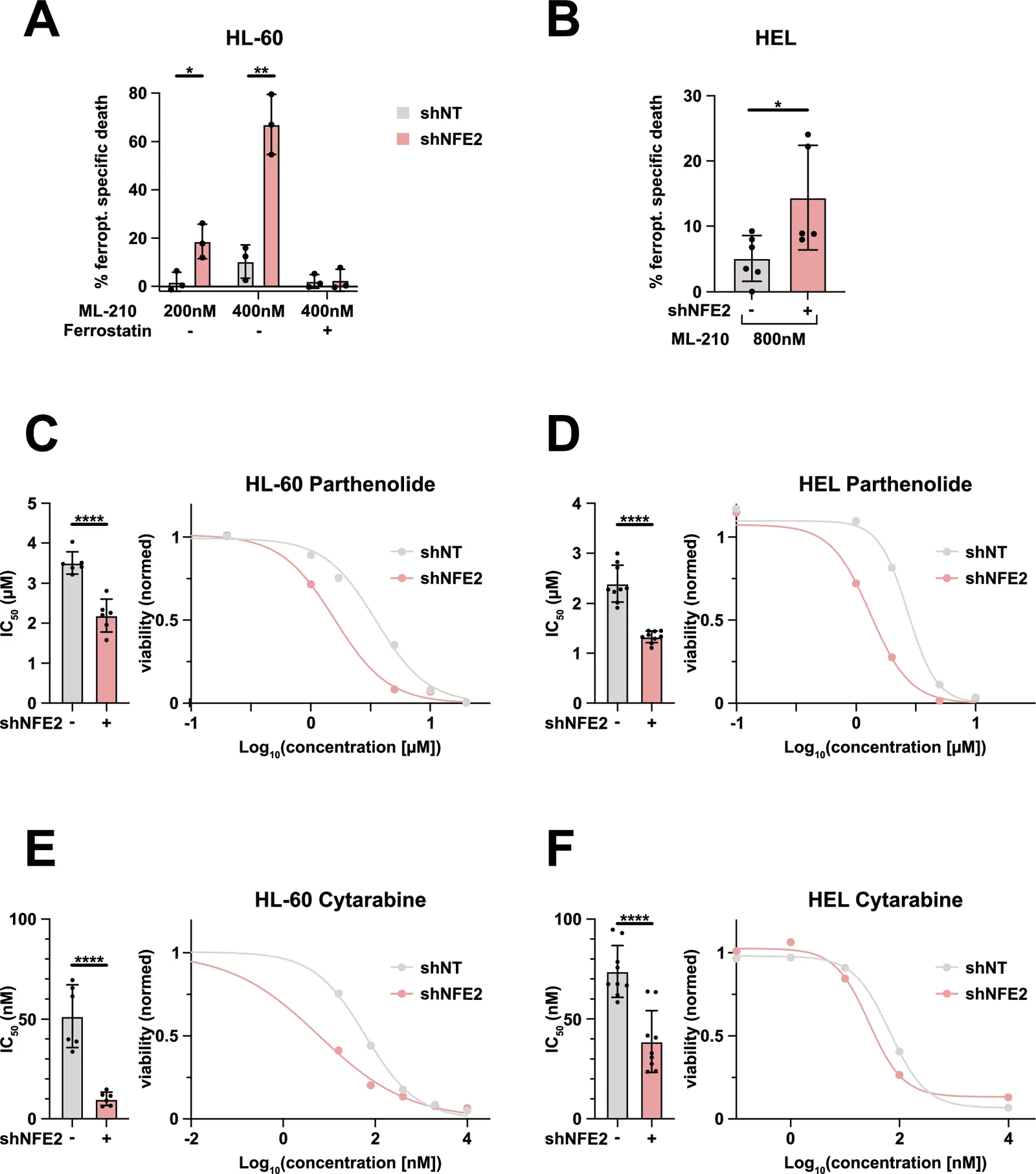
NFE2 depletion sensitizes AML cell lines to cytotoxic drugs and to the induction of ferroptosis. HL-60 (**A**, **C**, **E**) and HEL (**B**, **D**, **F**) cells were lentivirally transduced with a non-targeting shRNA (shNT) or an shRNA targeting NFE2 (shNFE2) vector. Cytotoxic treatments were initiated 48 h post-transduction. **A**, **B** Cells were treated with ML-210 for 24 h, and cell death was quantified by Annexin V/PI flow cytometry (*n* = 3 biological replicates for HL-60; *n* = 5-6 biological replicates for HEL). In HL-60 cells (A), ML-210 was tested with and without ferrostatin co-treatment to confirm ferroptosis dependence. Ferroptosis-specific cell death was calculated relative to vehicle controls, as described in the methods. Gating strategy described in Supplementary Fig. 9F. **C**–**F** For parthenolide and cytarabine treatments, viability was measured after 48 h using CellTiter-Glo, normalized to untreated controls, and IC₅₀ values were obtained by nonlinear regression in GraphPad Prism 10. Each panel displays the dose–response curve alongside the corresponding IC₅₀ bar chart. Representative dose–response curves are shown from one replicate per condition. **C**, **D** Parthenolide treatment: *n* = 6 biological replicates for HL-60; *n* = 9 biological replicates for HEL. **E**,** F** Cytarabine treatment: *n* = 6 biological replicates for HL-60; *n* = 9 biological replicates for HEL. **A**–**F** Mean depicted with standard deviation (SD). Statistical analyses were performed using two-sided, unpaired t-tests. *p < 0.05, **p < 0.01, ***p < 0.001, ****p < 0.0001.

Mechanistically, GSH depletion under oxidative pressure induces a distinct form of cell death called ferroptosis. Having demonstrated a crucial role for NFE2 both in maintaining intracellular GSH levels and in protecting leukemic cells from exogenous oxidative stress, we thus hypothesized that loss of NFE2 renders cells more susceptible to ferroptosis. To directly test this, we exposed HL-60 cells depleted of NFE2 as well as control cells to increasing concentrations of ML-210, a GPX4 inhibitor that induces ferroptosis by preventing the reduction of lipid peroxides. Consistent with our model, NFE2-depleted HL-60 cells displayed a significantly higher proportion of ferroptotic cell death than control HL-60s at identical concentrations of ML-210 (Fig. 6A). This effect was reversed by the addition of the ferroptosis inhibitor ferrostatin, demonstrating that cell death witnessed upon NFE2 depletion indeed occurs via ferroptosis (Fig. 6A). We next performed NFE2 depletion in another leukemic cell line, HEL, characterized by high NFE2 expression and low NRF2 protein levels (Supplementary Fig. 11). Like HL-60, NFE2-depleted HEL cells became more susceptible to ferroptosis induction via ML-210 (Fig. 6B). Cell death following exposure to a second inducer of ferroptosis and well-known glutathione depleter, parthenolide, was similarly increased in both HL-60 and HEL cells upon NFE2 depletion (Fig. 6C, D).

To confirm the relevance of NFE2 in preventing ferroptotic cell death, we reanalyzed two genome-wide CRISPR-activation screens investigating resistance to ferroptosis induction [22]. In SK-N-DZ cells treated with either RSL3 or Erastin, upregulation of NFE2 significantly suppressed ferroptosis (Supplementary Fig. 12A-D). Moreover, a CRISPR-knockout screen in K562 cells treated with formaldehyde, a GSH-scavenger [23], revealed NFE2 as a significant dependency (Supplementary Fig. 12E-H) [24]. Several different experimental approaches thus demonstrate a crucial role of NFE2 in preventing death by ferroptosis.

### NFE2 depletion renders AML cells more susceptible to chemotherapeutic agents

Cytotoxic drugs such as cytarabine (Ara-C), used for AML induction chemotherapy, exert their effect in part by producing reactive oxygen species (ROS). Vice versa, the inhibition of ferroptosis promotes Ara-C resistance in AML cells [25]. We therefore investigated the effect of NFE2 depletion on human AML cells exposed to chemotherapeutic agents. HL-60 and HEL cells depleted of NFE2 were significantly more sensitive to cytarabine than control cells (Fig. 6E, F). HEL cells also showed increased sensitivity to daunorubicin upon NFE2 depletion (Supplementary Fig. 13A and B).

### An NFE2 RedOx score predicts inferior survival in AML patients

**Fig. 7:**
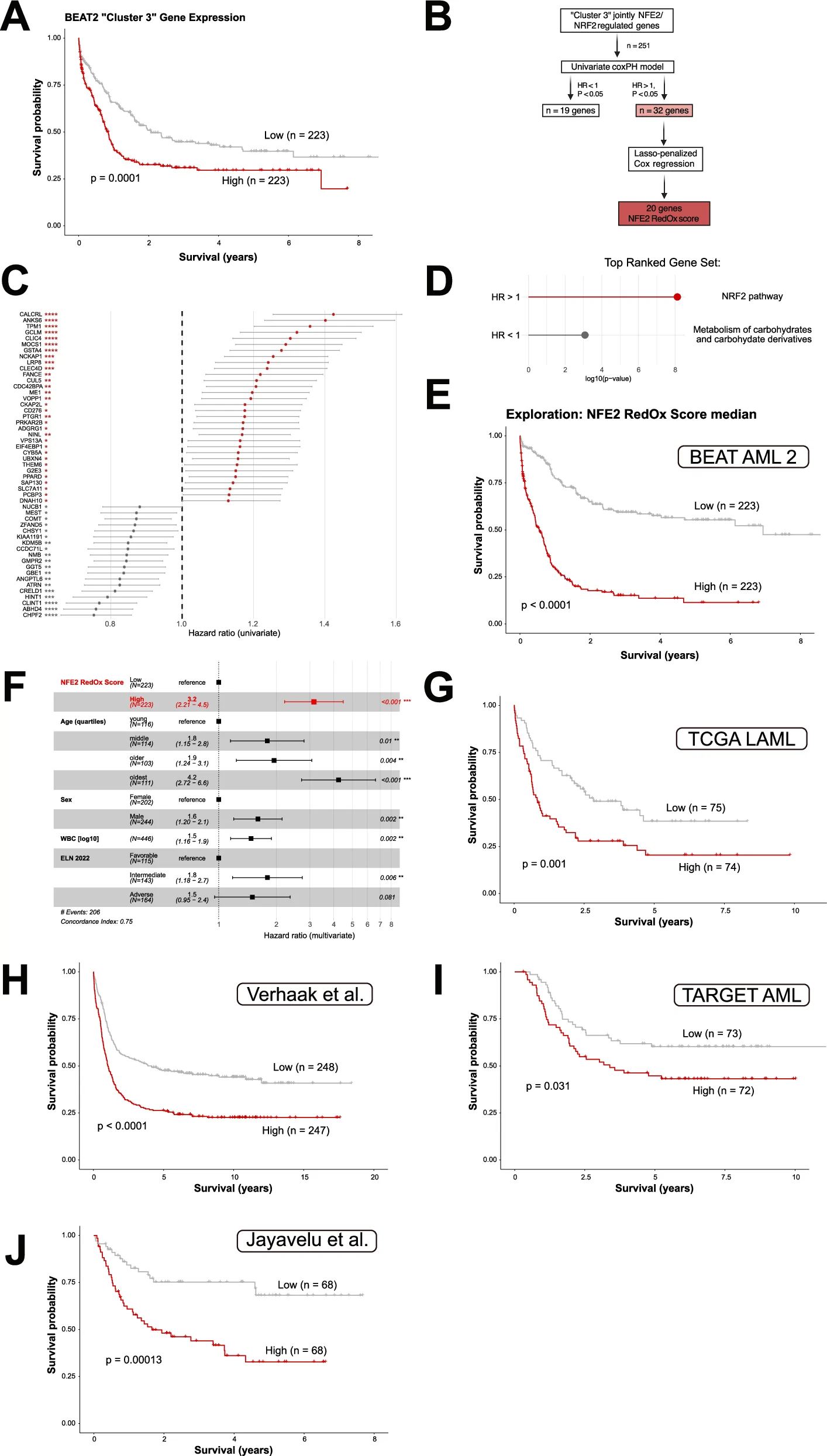
An NFE2 target gene signature predicts poor survival in AML. **A** Overall survival (OS) following outcome-agnostic stratification of diagnostic BEAT AML 2 samples using single-sample Gene Set Enrichment Analysis (ssGSEA) of the 251 genes assigned to cluster 3. **B** Schematic workflow for prognostic score derivation. Each “cluster 3” gene (Fig. 3) was tested individually for association with overall survival in BEAT AML RNA-seq samples using univariate Cox proportional hazards models (CoxPH). Genes with hazard ratio (HR) > 1 and *p* < 0.05 were retained and subjected to Lasso-penalized Cox regression, yielding a 20-gene NFE2 redox signature. Unless otherwise specified, analyses were restricted to *n* = 446 samples from initial diagnosis. **C** Forest plot of HRs with 95% confidence intervals (CIs) for predictive genes with *p* < 0.05 in univariate Cox analysis. p values from Wald tests: *p < 0.05, **p < 0.01, ***p < 0.001, ****p < 0.0001. **D** Top gene set enrichment (Metascape) result for genes from (B). Result for genes with HR > 1 in red, for genes with HR < 1 in gray. Bars indicate -log10(p) values. **E** Kaplan–Meier OS in BEAT AML diagnostic samples, comparing high (red) vs low (gray) NFE2 RedOx score groups (median split). The score was derived from a Lasso-penalized Cox model trained on this cohort. **F** Forest plot of HRs with 95% CIs from a multivariate CoxPH model in the BEAT AML cohort, including NFE2 RedOx score (median split) and clinical covariates (age, sex, white blood cell count (WBC), ELN 2022 risk group). **G**–**J** External validation in four independent cohorts: Kaplan–Meier OS for high (red) vs low (gray) NFE2 RedOx score groups (median split) in TCGA LAML (**G**), Verhaak et al. (**H**) [15], TARGET AML (**I**), Jayavelu et al. (**J**) [16]. Kaplan-Meier survival differences were analyzed with two-sided log-rank tests. HR and 95% CIs from CoxPH models were evaluated via Wald tests.

Our data strongly suggest that increased NFE2 activity will render AML cells more resistant to cytotoxic therapy and hence may lead to a decreased survival of AML patients. We tested this hypothesis by deriving a gene signature, consisting of novel NFE2 target genes with roles in redox regulation (Cluster 3 Fig. 3E, F). In a first analysis, we used all 251 genes in Cluster 3 and assigned an expression score to each newly diagnosed patient in the BEAT AML cohort. Higher expression of NFE2 antioxidant targets predicted worse overall survival (OS) in AML patients at diagnosis (Fig. 7A). We subsequently tested each of the 251 genes in Cluster 3 individually for significant association with overall survival at *p* < 0.05. This yielded 51 genes, 32 associated with poorer OS (hazard ratio (HR) > 1), the remainder with a better OS (Fig. 7B, Supplementary Table 7). Interestingly, NFE2 target genes associated with a poorer OS were highly enriched for stress response / NRF2 target genes, while the NFE2 targets associated with better OS were not (Fig. 7C, D). Genes with a HR > 1 and *p* < 0.05 were retained and subjected to Lasso-penalized Cox regression, yielding a 20-gene NFE2 RedOx signature (Fig. 7B).

In *n* = 446 patients from the BEAT AML cohort, using only samples taken at initial diagnosis, Kaplan-Meier survival analysis demonstrated that patients displaying expression of the NFE2 RedOx signature above the median (“high”) show significantly poorer OS compared to patients expressing levels below the median (“low”) (Fig. 7E, and Supplementary Fig. 14A). The signature retained its predictive power when patients were analyzed by quartiles (Supplementary Fig. 14B). In multivariate analysis, the NFE2 RedOx signature remained significant independently of known survival predictors including age, ELN classification and hyperleukocytosis (Fig. 7F, Supplementary Fig. 14C, *p* < 0.001, proportional hazards violation excluded, Supplementary Fig. 14D). Correspondingly, the RedOx score increases significantly across ELN risk categories (Supplementary Fig. 14E). Our NFE2 RedOx score also predicted significantly shortened OS in four independent validation cohorts (TCGA LAML, Verhaak et al., TARGET AML and Jayavelu et al.) [13–16] (Fig. 7G–J).

Our data thus suggest that identifying patients with high NFE2 RedOx signatures could aid in prognostic stratification. Moreover, lowering NFE2 activity may prove beneficial for ensuring therapeutic success in AML patients.

## Discussion

Leukemic transformation is associated with an increased ROS production [26, 27]. In a cohort of 49 AML patients encompassing many morphological subtypes (M1, M2, M4, and M5), 65% showed up to 100-fold greater levels of superoxide compared to healthy controls [28]. Superoxide overproduction increases H_2_O_2_ levels, promoting proliferation of AML cell lines as well as primary AML blasts [28].

Since ROS drive AML proliferation, thereby providing a competitive advantage and causing expansion of the malignant clone, their production is essential for leukemogenesis. On the other hand, if ROS levels exceed a certain threshold, cells experience “oxidative stress,” which damages cellular macromolecules, potentially causing cell death. ROS formation and antioxidative responses must consequently be carefully balanced. In addition to increased ROS production, AML cells therefore also show augmented antioxidative responses. For example, AML cells express elevated levels of multiple glutathione pathway regulators [29]. To date, the transcription factor NRF2 has been considered the master regulator of genes encoding antioxidant response proteins such as the glutathione regulators SLC7A11, GCLM, and GPX4 [30]. We show here that these genes constitute novel NFE2 targets (Supplementary Fig. 8). Importantly, the presence of NFE2 confers chromatin accessibility at these loci beyond that caused by NRF2 alone, resulting in increased gene expression. Moreover, presence of NFE2 significantly increased glutathione levels (Fig. 4A, B). Contrary to a previous report, which proposed antagonistic functions of the two transcription factors [21], we show that NFE2 and NRF2 cooperate in providing redox protection. Mechanistically, we thus demonstrate for the first time that NFE2 positively regulates the expression of several key antioxidant enzymes and controls GSH levels in AML cells.

NFE2 constituted one of the most significant redox dependencies in MLL-AF9 transformed leukemic cells subjected to prolonged H_2_O_2_ exposure (Fig. 5). Importantly, dependency on NFE2 was much more pronounced than dependency on NRF2, which has recently been proposed as a target for overcoming resistance of AML stem cells [31]. GSH is essential for the reduction of H_2_O_2_ to H_2_O via GSH peroxidases (GPX) [32]. We therefore propose that cell death in the absence of NFE2 is caused by reduced expression of the novel NFE2 target genes identified in this study, resulting in insufficient GSH levels. These data underscore the novel and central role for NFE2 in protecting leukemic cells from ROS-mediated cell death.

Death caused by oxidative stress and the resulting lipid peroxidation has been recognized as a distinct form of cellular demise, termed ferroptosis. Protection from ferroptosis requires adequate glutathione levels. These ensure the activity of glutathione peroxidase 4 (GPX4), a key detoxifying enzyme which catalyzes phospholipid hydroperoxide reduction, thereby preventing their accumulation in cell membranes, which would result in ferroptosis. Because of their elevated ROS levels, AML cells are particularly vulnerable to ferroptosis. Compared to their healthy counterparts, primary AML cells are more sensitive to GPX4 inhibition [33] and to GSH depletion, induced by agents such as parthenolide [29] or by cysteine starvation [34]. Huang and colleagues have recently demonstrated that the leukemogenic potential in an MLL-AF9-driven murine model depends critically on the maintenance of low redox levels [35]. In several murine AML models, decreasing GSH levels though either genetic or pharmacologic inhibition of key enzymes and transporters increased ROS levels and attenuated leukemic progression resulting in improved survival [29, 36, 37]. Moreover, low ROS levels confer resistance to cytarabine, one of the backbones of AML induction chemotherapy. Here we demonstrate that shRNA-mediated depletion of NFE2 sensitized AML cells to ferroptosis induction by ML210 (Fig. 6A, B), to GSH-depletion by parthenolide (Fig. 6C, D), and to cytotoxicity by cytarabine (Fig. 6E, F).

Cysteine constitutes the rate-limiting substrate in glutathione synthesis, and its cellular import depends critically on activity of the SLC7A11 cystine/glutamate antiporter. As cysteine is required to prevent ferroptosis in AML cells [34], its depletion exploits the metabolic vulnerabilities of leukemic cells [32]. Fittingly, SLC7A11 has recently been identified as a novel AML dependency and its pharmacological inhibition synergized with daunorubicin and cytarabine in a xenograft model of primary human NPM1-mutated AML [38]. Here we demonstrate that SLC7A11 constitutes a novel NFE2 target gene (Fig. 1G), mechanistically explaining increased cytarabine cytotoxicity in NFE2-depleted cells.

Augmented NFE2 activity in leukemic cells therefore leverages several coordinated mechanisms to enhance cellular resistance to oxidative stress: i) increased uptake of critical educts, ii) increased glutathione synthesis and replenishment as well as iii) increased activity of detoxifying enzymes. Targeting the elevated NFE2 levels in AML would thus impair redox resistance through several vulnerabilities simultaneously, possibly circumventing the development of resistance mechanisms.

I. Decreasing expression of the SLC7A11 cystine/glutamate antiporter, essential for uptake of the rate-limiting substrate cysteine.
II. Decreasing expression of the rate limiting enzyme in glutathione synthesis, glutamate-cysteine ligase (GCL), whose subunit GCLM constitutes a novel NFE2 target gene.
III. Decreasing expression of glutathione peroxidase 4 (GPX4), which catalyzes the reduction of phospholipid hydroperoxides, thereby preventing ferroptosis.

To assess the clinical impact of NFE2 antioxidant target gene expression, we derived a score composed of 20 novel NFE2 targets. In five independent AML patient cohorts we show that individuals with a high NFE2 RedOx score show significantly worse OS. Our data therefore suggest that redox protection through elevated NFE2 target gene expression confers a more aggressive AML phenotype. Targeting NFE2 activity in AML cells may thus revert this advantage, increase sensitivity to chemotherapy via induction of ferroptosis and thereby induce deeper remissions.

## Supplementary information

Supplemental Material

Supplemental Table 1

Supplemental Table 2

Supplemental Table 3

Supplemental Table 5

Supplemental Table 6

Supplemental Table 7

## Data Availability

All data generated or analyzed during this study are either included in this published article and its supplementary information files or will be made available from the corresponding authors upon request. The RNA-seq, ChIP-seq and ATAC-seq data generated during the current study have been submitted to the GEO database under the accession numbers GSE311575, GSE311576, and GSE311577.
