# Supplementary material for: A novel role for transcription factor NFE2 in redox regulation and chemotherapy resistance in acute myeloid leukemia
Source: Leukemia. 2026 Aug 19;40(11):2481–93. doi: 10.1038/s41375-026-03095-x (PMC13713581; doi:10.1038/s41375-026-03095-x)
Supplement: Supplementary file 1 [file 41375_2026_3095_MOESM1_ESM.pdf]

A novel role for transcription factor NFE2 in redox regulation and chemotherapy resistance in acute myeloid leukemia

Anne Marie Staehle, Florian Perner, Hans Felix Staehle, Katharina Gellrich, Fiona Preiß, Annika Grafemeyer, Jana Schulze, Jean-Luca Funk, Albert Gründer, Julia Berner, Sander Bekeschus, Christoph Koellerer[#] and Heike L. Pahl[#]

Supplemental Methods, Tables, Figures & References

Supplemental Methods

Cell Lines

HEL and HL-60 cells were obtained from DSMZ (Braunschweig, Germany). CB3 cells, an NFE2-KO murine erythroleukemia cell line immortalized by Friend murine leukemia virus mediated insertional activation of Fli1 and loss of p53, were a kind gift of V. Blank and E. Bresnick.[1] Cell lines were cultivated in the recommended media in suspension cell culture flasks or 96-well plates at 37°C in a 5% CO_2 atmosphere. To maintain optimal culture conditions, the cells were passaged every 24 h to 48 h. Authentication of human cell lines was performed by the DSMZ. Cell lines were tested negative for Mycoplasma.

Flow cytometry

Flow cytometry experiments were performed on a BD FACS Fortessa. GFP and mCherry signals were determined to assess transduction efficiency. Cellular levels of reactive oxygen species (ROS) were measured by staining with CellROX Deep Red (Thermo Fisher Scientific, Waltham, MA, USA) Cellular levels of reduced glutathione (GSH) were measured by staining with 5mM monochlorobimane (mBCI, Thermo Fisher Scientific) for 10min. Cell death was measured using the Pacific Blue™ Annexin V Apoptosis Detection Kit with PI (Biolegend, San Diego, CA, USA). To isolate viable transduced CB3 cells for joint ATAC-seq and RNA-seq analysis, cells were sorted using either 7-AAD or DAPI (BioLegend).

Lentiviral Modulation of NFE2 and NRF2 activity

A pLeGO-iC2 (termed iC2) and a modified pLeGO-iG-U6 vector (termed iG) were used for lentiviral transduction as previously described.[2,3] The iC2-NFE2, iG-NFE2 and iG-NRF2_Neh2_AM vectors (**Suppl.Fig 4A**) were used for expression of NFE2 and NRF2. While iC2-NFE2 and iG-NFE2 contain a wt NFE2 cDNA, iG-NRF2_Neh2_AM contains a cDNA encoding an AM-tagged, Neh2-mutated NRF2 (**Suppl.Fig 4A**). Empty vectors (iC2-empty and

iG-empty) were used as controls. To study NFE2 depletion, an NFE2 shRNA or a non-targeting control shRNA were introduced into HL-60 and HEL cells using the iG-shNFE2 (5'-GGATTATCCCTCAACTATA-3')[3] or the iG-shNT (5'-GGGTGAACTCACGTCAGAA-3')[4] constructs.

tert-Butylhydroperoxid (tBHP) treatment of HL-60 cells

Cells were treated with vehicle or 100 μM tert-Butylhydroperoxid (tBHP, Thermo Fisher Scientific) for 4 hours and harvested for RNA isolation and RTqPCR.

H_2O_2 treatment of HL-60 cells

Cells were treated with vehicle or 50 μM H_2O_2 once daily for 48h. Cell death was assessed via AnnexinV-PI (BioLegend) flow cytometry and the percentage of H_2O_2 -specific cell death was calculated using the following formula:

$$\%\ \mathrm{H_2O_2}\ -\text{specific cell death} = \frac{\%\text{AnnexinV}^{-}\text{PI}^{-}\ (\text{veh})\ - \%\text{AnnexinV}^{-}\text{PI}^{-}\ (\text{treated})}{\%\text{AnnexinV}^{-}\text{PI}^{-}\ (\text{veh})}$$

Cytarabine, daunorubicine and parthenolide treatments

48h after transduction with iG-shNT/shNFE2, cells were seeded into 96-well plates and treated with different concentrations of cytarabine, daunorubicine or parthenolide (all purchased from MedChemExpress, Monmouth Junction, NJ, USA) for 48h. Cells were then harvested, and the CellTiter-Glo® Luminescent Cell Viability Assay (Promega, Fitchburg, WI, USA) was performed according to manufacturer's instructions. Luminescence was detected with a Tecan Infinite M200 microplate reader (Tecan, Maennedorf, Switzerland) and IC_{50} values were calculated using non-linear regression in GraphPad Prism 10.

Ferroptosis assay

Cells were treated with vehicle or different concentrations of the ferroptosis inducer ML-210 for 24h (MedChemExpress). Inhibition of ferroptosis was achieved by simultaneous treatment with 10mM Ferrostatin (Merck, Darmstadt, Germany). Cell death was assessed via AnnexinV-PI (BioLegend) flow cytometry, and the percentage of ferroptosis-specific cell death was calculated using the following formula:

$$\%\ \text{ferropt.} - \text{specific cell death} = \frac{\%\text{AnnexinV}^{-}\text{PI}^{-}\ (\text{veh}) - \%\text{AnnexinV}^{-}\text{PI}^{-}\ (\text{treated})}{\%\text{AnnexinV}^{-}\text{PI}^{-}\ (\text{veh})}$$

Genome-wide CRISPR-Cas9 screening:

Murine hematopoietic stem and progenitor cells (sorted for: Lin-/Kit+/Sca1+) were immortalized using the MLL-AF9 (KMT2A::MLLT3) oncogene as previously described.[5] Cells were cultured and expanded in DMEM + 20% FBS supplemented with myeloid cytokines (IL3+IL6+SCF, all at 25ng/ml) to a total number of 500 Mio cells. Subsequently, cells were transduced with lentiviral particles to express the M1 “Liu Mouse CRISPR Knockout Library” in the lentiCRISPR v2-Blast vector system (Addgene Pooled Library #1000000173) at a low multiplicity of infection (MOI). This facilitated the expression of 92,919 sgRNA plasmids for the knockout of all protein coding genes in the genome with 5 sgRNAs each along with the Cas9 protein.

After selection with Blasticidine (10μg/ml) for 5 days, library-transduced cells were subjected to functional genomic screening for redox mediators. To this end, 35 Mio cells were harvested for assessment of the baseline frequencies of sgRNA barcodes and triplicates of 35 Mio cells were treated with either H_2O_2 or PBS as a control for 10 days. To create a culture environment simulating repeated redox stress, the cultures were challenged once daily with 100μmol of H_2O_2 or PBS. After 10 days, cells were harvested, washed once in PBS and genomic DNA was extracted using the phenol-chloroform method. CRISPR-Amplicon libraries for sequencing

of sgRNA barcodes were generated as previously described.[6,7] Biocomputational quantification and evaluation of differential sgRNA frequencies between the conditions was performed using the MaGECK pipeline.[8,9]

RNA Extraction and Quantitative real-time PCR (qRT-PCR)

RNA isolation and reverse transcription were performed using RNAeasy (QIAgen, Venlo, Netherlands) and SuperScript™ II Reverse Transcriptase (Invitrogen, Thermo Fisher Scientific). In order to determine gene expression, the resulting cDNA from CB3, HL-60 or HEL cells was assayed by quantitative PCR using either TaqMan Probes (**Suppl. Table 8**) or PowerUp SYBR Green Master Mix (Thermo Fisher Scientific, primers see **Suppl. Table 9**). Murine or human β-2-microglobulin was used as a housekeeping gene. Data were analyzed using the ΔΔCT method.

Western blotting

Cells were lysed in RIPA buffer (150mM NaCl, 1% Triton X-100, 0.5% NaDoc, 0.1% SDS and 50mM Tris, pH 8.0) for 30 minutes on ice. Cell debris was removed by centrifugation. Prior to western blotting, protein concentrations were estimated by Lowry assay (Bio-Rad Laboratories, Hercules, CA, USA). The following primary and secondary antibodies were used: anti-NFE2 (HPA001914, Atlas Antibodies, Stockholm, Sweden), anti-NFE2 (66436-1-Ig, Proteintech, Rosemont, IL, USA), anti-NRF2 (ab62352, Abcam, Cambridge, UK), anti-NRF2 (#12721, Cell Signaling Technology, Danvers, MA, USA), anti-GAPDH (G8795, Thermo Fisher Scientific), anti-AM (91111, Active Motif, Carlsbad, CA, USA), anti-Actin (A5441, Merck), anti-rabbit IgG HRP (#7074, Cell Signaling Technology), anti-mouse IgG HRP (NA931V, GE Healthcare, Chicago, IL,USA).

Analysis of the NFE2 interactome

Plasmids

Human NFE2 cDNA, fused to humanized BirA or "miniTurbo BirA" with a coding linker, was cloned into the lentiviral pLeGO-iG2 expression vector. [10–12]

Lentiviral transduction and biotinylation

CB3 cells were transduced with either, pLeGO-iG2-NFE2-LC-BirA-HA or pLeGO-iG2-NFE2-LC-miniTurbo. 24 hours post transduction, GFP positive cells were incubated with 50μM biotin (Cat: B4639 Sigma-Aldrich) for 20 min (miniTurbo) or 6 h (hBirA) and subsequently lysed.

Biotin-Streptavidin pulldown

Streptavidin sepharose beads (Cat: 17-5113-01, GE Healthcare) were used to capture biotinylated proteins, biotin-saturated beads were used as a negative control. To minimize nonspecific signals resulting from DNA-coupled proteins, ethidium bromide (Cat: 15585011, Thermo Fisher Scientific) was added. Subsequently, beads were harvested and used for on-bead digestion with trypsin.

Mass Spectometry

Tryptic peptides were analyzed by nanoflow-LC-MS/MS with a high performance nanoflow-HPLC Orbitrap based ms/ms system (Thermo Fisher Scientific). The raw data files were analyzed with MaxQuant software (version 1.5.3.30) using Uniprot murine FASTA database.[13,14] Label-free quantification values (LFQ) and match between run options were selected. An intensity based absolute quantification (iBAQ) algorithm was used for subsequent relative protein abundance estimation.[15] The obtained protein files were analyzed by Perseus software (MQ package, version 1.6.12), in which contaminants and reverse hits were filtered out.[14] Protein identification based on non-unique peptides as well as proteins identified by only one peptide in the different triplicates were excluded to increase protein prediction accuracy.

For identification of the bait interactors, LFQ intensity-based values were transformed on the logarithmic scale (log2) to generate a Gaussian distribution of the data. This allowed the imputation of missing values based on the normal distribution of the overall data (in Perseus, width = 0.3; shift = 1.8). The normalized LFQ intensities were compared between grouped GFP triplicates and non-GFP triplicates, using 1% as the permutation-based false discovery rate (FDR) in a two-tailed t-test. The threshold for significance (S0), based on the FDR and the ratio between biotin pulldown and control samples was kept at the constant value of 2. Relative abundance plots were obtained by comparison of the iBAQ values of NFE2 interactors. The values of the control sample iBAQ values were subtracted from the corresponding proteins in the biotin pull-down and were next normalized on the bait protein for scaling and data representation purposes.

RNA Sequencing (RNA-Seq)

CB3 cells were gated for viability, sorted by fluorescent markers, lysed and total RNA was extracted. For the first batch of samples (CB3 transduced with empty/ R273W/ Del297-300/ wt NFE2/ K287R, Fig. 1) cells were lysed using TRIzol (Invitrogen, Thermo Fisher Scientific) per manufacturer's instructions and sequencing was performed on a HiSeq3000 (Illumina, San Diego, CA, USA) with 75bp read length. For the second batch (CB3 transduced with empty/ NFE2/ NRF2 / NFE2+NRF2, Fig. 3) the RNeasy Mini Kit (Qiagen) was used for extraction followed by sequencing on a NovaSeq (Illumina) for 100 cycles. In both cases, RNA quality was assessed by capillary electrophoresis, libraries were created using the Illumina stranded mRNA Prep kit and sequencing was performed in paired-end mode.

Chromatin Immunoprecipitation (ChIP-seq)

ChIP-seq was performed as previously described.[16,17] Immunoprecipitation was performed with the following antibodies: NFE2 (GTX102698, GeneTex, Irvine, CA, USA), H3K4me3

(C15410003, Diagenode, Seraing, Belgium), H3K27ac (C15410196, Diagenode). Libraries were sequenced on a HiSeq3000 in paired-end mode with a read length of 75 bp.

For ChIP-Seq against NRF2, this protocol was modified. CB3 cells transduced with an AM-tagged, Neh-2-mutated NRF2 construct were fixed using 2mM DSG (Disuccinimidylglutarat, Sigma-Aldrich, Merck) and 1% formaldehyde. Shearing was performed according to the NEXSon protocol[17] and immunoprecipitation with an anti-AM antibody (91111, Active Motif) was performed using the iDeal Transcription Factor ChIP-Seq kit (Diagenode). Libraries were prepared using the NEBNext Ultra II DNA Library Prep Kit (NEB, Ipswich, MA, USA) and sequenced on a NovaSeq (Illumina) in paired-end mode for 100 cycles.

Assay for Transposase-Accessible Chromatin (ATAC-seq)

We performed Omni-ATAC as previously described.[18] In short, 10.000-50.000 viable CB3 cells were sorted, lysed and tagmented at 37°C for 30 minutes. Libraries were quality-controlled for proper enrichment and fragment size distribution, then sequenced in paired-end mode. The first batch of samples (CB3 transduced with NFE2, Fig. 2) was sequenced on a HiSeq3000 (Illumina) with a read length of 75 bp. The second batch (CB3 transduced with NFE2/NRF2, Fig. 3) was sequenced for 100 cycles on an Illumina NovaSeq instrument.

RNA-Seq Data Analysis

Raw fastq files were trimmed using Trim Galore! (v0.6.10, Babraham Institute) with default parameters. Salmon (v1.10.3)[19] was used for quantification of transcripts. In short, a decoy-aware salmon index was created from the GRCm39 primary assembly and the protein coding transcript sequences from GENCODE (vM34) including eGFP and mCherry sequences. Transcripts were counted with the following parameters („--seqBias --gcBias“).

The resulting transcript-level count matrices were loaded with the R package tximport (v1.36.0)[20] to create gene-level counts that were pre-filtered for meaningful gene expression levels. Differential gene expression was quantified using DESeq2 (v1.48.0)[21] using a batch-

aware design formula and Wald-testing followed by Benjamini-Hochberg (BH) procedure. The lfcShrink() function with the „ashr“ parameter was used to adjust inflated log2 fold-change values at lower expression levels.

Gene set enrichment (GSEA) analyses were conducted by ranking genes according to their DESeq2 test statistic and applying the fGSEA (v1.34.2, https://doi.org/10.1101/060012) implementation in the clusterProfiler R package (v4.16.0).[22] When analyzing the enrichment of specific smaller groups of genes we used metascape[23] for functional annotation.

For more complex gene-level clustering tasks (Fig. 3), hierarchical clustering was replaced with a custom consensus clustering. Briefly, we ran 1000 bootstrap iterations of k-means with a fixed k, each time sampling 80% of genes and accumulated a gene*gene co-clustering matrix (fraction of times two genes co-assigned). We then performed hierarchical clustering on this matrix, cutting the dendrogram to k clusters. The k with the cleanest separation and most informative clusters was selected. All RNA-seq gene-level analyses were performed using R (v4.5.0) and RStudio (2024.12.1).

ChIP-seq Data Analysis

ChIP-seq analyses were performed as previously described[24] and reads were mapped to mm10.

ATAC-seq Data Analysis

Raw fastq files were trimmed using Trim Galore! (v0.6.10, Babraham Institute) with default parameters. Alignment to mm10 was performed using bowtie2 (v2.5.4)[25] with the following parameters „-I 0 -X 2000 --very-sensitive“. Sambamba (v1.0.1)[26] was used to remove duplicate and improperly mapped reads and to filter for canonical chromosomes only.

The filtered bam files were submitted to +4/-5 shifting to better reflect Tn5 binding sites using the alignmentSieve() function from the deeptools (v3.5.5)[27] suite. For peak calling we used the current implementation of the ENCODE ATAC-Seq pipeline (https://github.com/ENCODE-

DCC/atac-seq-pipeline). In short, we isolated 1bp Tn5 insertion sites and used these as an input for the MACS2 (v2.2.9.1)[28] callpeak() function with the following parameters „--format BED --gsize mm --keep-dup all --shift -75 --extsize 150 –nomodel". Peaks were blacklisted by combining the ENCODE blacklist[29] of high signal regions with a blacklist for high homology to mtDNA. Importantly, the ENCODE blacklist at the Mafg locus was lifted in Fig. 2 since that locus displayed differential ATAC/RNA signal between conditions and close inspection revealed no signal artifacts. Coverage files were generated using deeptools bamCoverage with CPM normalization and were blacklisted as well. Whenever necessary mean values of coverage scores were aggregated for each condition using wiggletools (1.2.11). [30]

Heatmaps and profiles of coverage across genomic coordinates were generated using the deeptools plotProfile() and plotHeatmap() functions. In order to test for differentially accessible regions (DARs), we counted Tn5 insertion events in 50bp sliding windows across called peaks using the R bioconductor package csaw (v1.42.0,).[31] We then used edgeR (v4.6.1, PMID: 39844453) to fit a generalized linear model and used the edgeR implementation of quasi-likelihood F-tests to identify DARs. After testing, neighboring windows within one nucleosome (150bp) were merged and their test statistic adjusted using the csaw mergeResults() function. Motif usage analysis was performed by using the package chromVAR (v1.30.0)[32] according to its vignette. The motif databases were JASPAR2024[33] as well as a custom database of clustered motifs (https://github.com/vierstralab/motif-clustering). We used the iterative clustering method[34] to create uniformly sized 500bp peaks as input into chromVAR. Operations on genomic coordinates were performed using the GenomicRanges object class from Bioconductor (v1.60.0)[35] and all ATAC counts analyses were performed using R (v4.5.0) and RStudio (2024.12.1).

Survival Analysis in the BEAT2 AML Cohort

Raw RNA-seq counts and clinical summary were downloaded from the official BEAT2[36] repository (https://biodev.github.io/BeatAML2/). To better integrate the data, we performed

batch-correction across samples using the POIBM method (v0.9.0)[37]. The batch-corrected counts were then variance-stabilized using the DESeq2 vst() function, and used as input for single-gene univariate Cox models to estimate hazard ratios and subsequently for LASSO regression. More specifically, for generation of the NFE2 RedOx score, we fit a regularized Cox regression model using the R package glmnet (v4.1-8)[38] with alpha = 1 (LASSO) and ten-fold cross-validation. The model selected at a penalty λ_min = 0.0219 provided the coefficients which constitute our NFE2 RedOx score.

Survival analyses were performed selecting only samples at AML diagnosis from the BEAT2 cohort; for TCGA LAML[39], Verhaak *et al.,*[40] TARGET AML[41] and Jayavelu *et al.*[42] we lacked this clinical information. In short, the R package „survival“ (v3.8-3) was used to fit Cox proportional hazards (coxPH) and Kaplan-Meier (KM) models to the BEAT2 data. For the main CoxPH model, next to our score we added the covariates „age“ (split into quartiles), „sex“, „WBC counts“ (log10-scaled) and the ELN classification. This information is found in the BEAT2 clinical summary linked above. In order to plot hazard ratios and survival curves, we used the plotting functions provided by the R package survminer (v0.5.0).

Statistical analysis

Two-tailed, unpaired Student's t-tests were used to determine whether a significant ($p<0.05$) difference existed between two groups using the GraphPad Prism 10 software, unless otherwise stated.

Public Datasets Used in This Study

The following NFE2 and NRF2 ChIP-Seq datasets were used: ENCODE project: ENCSR000FCC (NFE2 in K562), ENCSR552YGL (NFE2-eGFP in K562), ENCSR485GKE (NRF2 in K562), ENCSR197WGI (NRF2 in IMR90), ENCSR488EES (NRF2 in HepG2), ENCSR584GHV (NRF2 in A549), ENCSR707IUN (NRF2 in HeLa-S3); Nrf2 ChIP-seq in murine macrophages,[43] available under accession PRJDB4070 in the Japanese DDBJ

database; Nrf2 ChIP-seq in oesophageal tissue[44] from Nrf2−/−, WT, and Keap1−/− mice, available under GEO accession GSE122504.

The following AML datasets were used: BEAT2,[40] TARGET AML,[41] TCGA LAML,[39] Verhaak *et al.*,[40] Jayavelu *et al.*.[42]

The following CRISPR-Screen datasets were accessed from BioGRID ORCS (https://orcs.thebiogrid.org/): Alborzinia *et al.*,[45] Zhao *et al.* [46]

NFE2 gene expression data was accessed from the DepMap Portal: DepMap, Broad (2026). DepMap Public 26Q1. Dataset. depmap.org.[47]

Suppl. Tables 1,2,3,5,6 and 7: see separate excel files.

Suppl. Table 4

Ranking	Protein name	Gene names	Mean Log2FC
1	ATPase family AAA domain-containing protein 3	Atad3	3,38308333
2	Sentrin-specific protease 3	Senp3	3,09796667
3	Zinc finger CCCH domain-containing protein 18	Zc3h18	2,59461667
4	Small ribosomal subunit protein eS27	Rps27	2,28663333
5	Tubulin beta-4B chain; Tubulin beta-4A chain; Tubulin beta-2A chain	Tubb4b;Tubb4a;Tubb2a	2,17345
6	YTH domain-containing family protein 2	Ythdf2	1,95441667
7	Heterogeneous nuclear ribonucleoprotein L-like	Hnrnpll	1,83528333
8	Tubulin beta-5 chain	Tubb5	1,69626667
9	BAG family molecular chaperone regulator 2	Bag2	1,64861667
10	Fibulin-1	Fbl	1,46566667
11	Histone deacetylase 1	Hdac1	1,39731667
12	Solute carrier family 25 member 3	Slc25a3	1,20611667
13	Large ribosomal subunit protein uL14	Rpl23	1,19451667
14	Heterogeneous nuclear ribonucleoprotein F	Hnrnpf	1,1907
15	Small ribosomal subunit protein uS11	Rps14	1,12346667
16	Small ribosomal subunit protein eS26	Rps26	1,03535
17	Large ribosomal subunit protein eL24	Rpl24	0,97808333
18	Nucleolar protein 56	Nop56	0,9461
19	Heat shock cognate 71 kDa protein	Hspa8	0,85786667

Suppl. Table 4: Proteins identified as interacting with NFE2 by biotin-streptavidin pulldown using two different mass spectrometry approaches (see Suppl. Fig. 6)

Suppl. Table 8

Gene Expression Assay	Description
Nqo1	Applied Biosystems, Mm01253561_m1
GCLM	Applied Biosystems, Hs00978072_m1
NFE2	Forward Primer: 5’ GGC AGG AGA TCA TGT CCA TCA 3’
	Reverse Primer: 5’ GGT GGT GGA GGT CCA AGG TAT G 3’
	TaqMan probe: 6FAM-AGC TGC AGG GTC TG-MGBNFQ
mB2m	Forward Primer: 5’ CTT TCT GGT GCT TGT CTC ACT GAC 3’
	Reverse Primer: 5’ GGT GGC GTG AGT ATA CTT GAA TTT G 3’
	TaqMan probe: 6FAM-ATC CAG AAA ACC CC-MGBNFQ
hB2M	Forward Primer: 5’ TCT TTC TGG CCT GGA GGC TAT C 3’
	Reverse Primer: 5’ TGC TGG ATG ACG TGA GTA AAC C 3’
	TaqMan probe: 6FAM-AGC GTA CTC CAA AGA T -MGBNFQ

Suppl. Table 8: Primers and TaqMan probes used for RTqPCR.

Suppl. Table 9

Target	Primers
SLC7A11	Forward Primer: 5’ TGT GCT GAC AAA TGT GGC CT 3’
	Reverse Primer: 5’ ATT TCC CAG TAG CCG CTC AG 3’
OSGIN1	Forward Primer: 5’ ACT GGA TGC AGA AGA AGC GAA 3’
	Reverse Primer: 5’ CCA GAC CCT TCT TGA CCA CG 3’
hB2M	Forward Primer: 5’ TCT TTC TGG CCT GGA GGC TAT C 3’
	Reverse Primer: 5’ TGC TGG ATG ACG TGA GTA AAC C 3’

Suppl. Table 9: Custom primers used for RTqPCR with SYBR Green.

Suppl. Fig.1

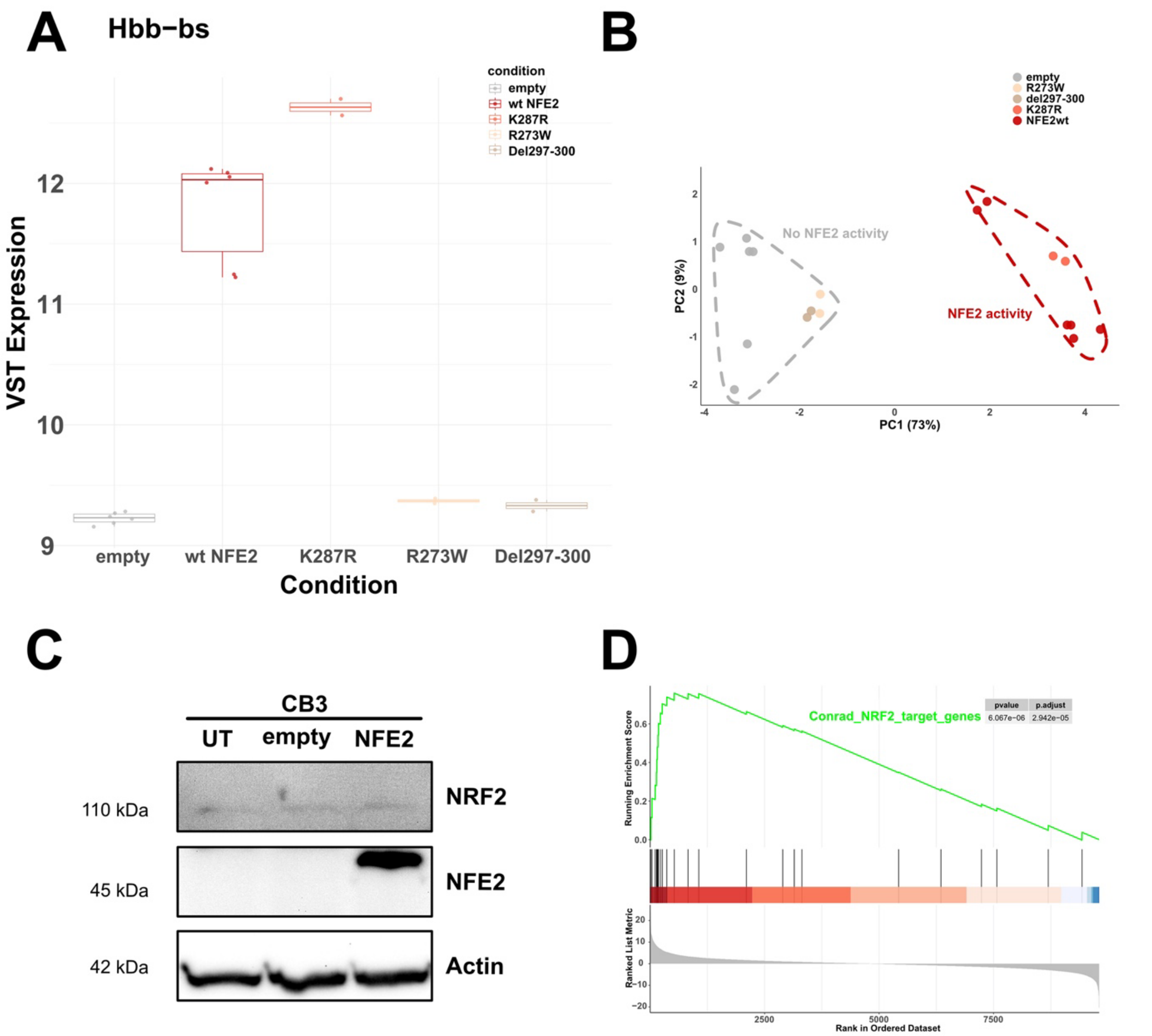

Suppl. Fig. 1: Additional characterization of transcriptional changes induced by NFE2 overactivity
(A) VST-normalized RNA-seq counts for the gene encoding hemoglobin subunit β (Hbb-bs) across the following conditions in CB3 cells: Empty (n = 6 biological replicates), wt NFE2 (n = 6 biological replicates), K287R (n = 2 biological replicates), R273W (n = 2 biological replicates), Del297-300 (n = 2 biological replicates). Box plot elements: Median, IQRs, max. whisker extension = 1.5 IQR. **(B)** PCA plot of VST-transformed expression using the 200 most variable genes. **(C)** Western Blot of untransduced (UT) CB3 cells and CB3 cells transduced with either an empty vector or a vector expressing wt NFE2. Primary antibodies against NRF2 (Cell Signaling Technology), NFE2 (Atlas Antibodies) and beta-actin were used. **(D)** GSEA enrichment of NRF2 target genes (Conrad *et al.*) in the CB3 transcriptome ranked by differential expression between NFE2-active and NFE2-inactive conditions.

Suppl. Fig. 2

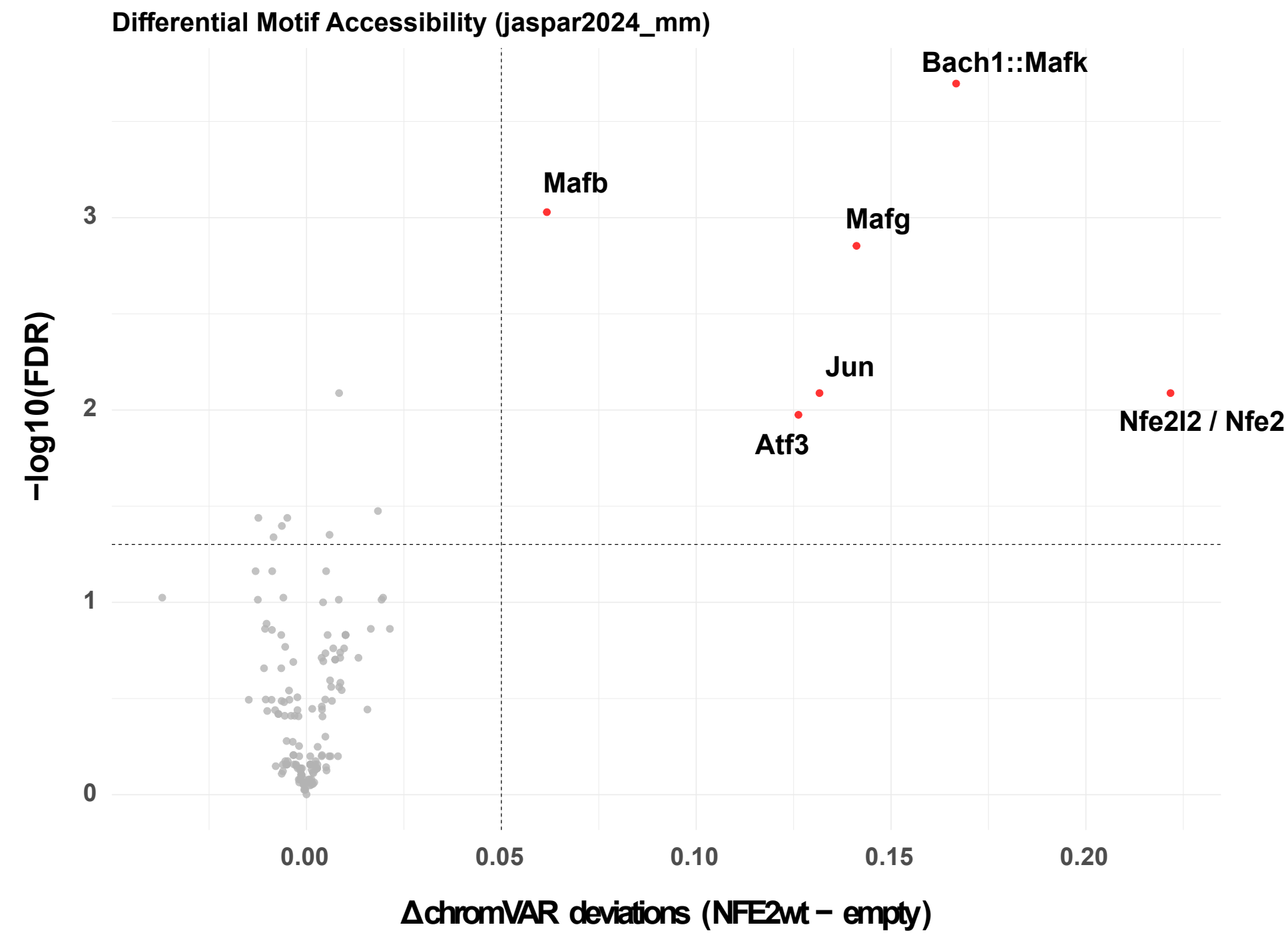

Suppl. Fig. 2: Motif usage in NFE2-overexpressing cells (JASPAR 2024)
ChromVAR TF motif deviation scores between NFE2-overexpressing and empty control CB3 cells (n = 3 biological replicates per condition). The motifs from the murine JASPAR 2024 database were scored on a consensus set of 500 bp-wide ATAC peaks. We have expanded the name from motif MA0150.3 from “Nfe2l2” to “Nfe2l2 / Nfe2” for clarity. Two-sided unequal variances t-test was used to evaluate statistical significance, followed by BH correction.

Suppl. Fig. 3

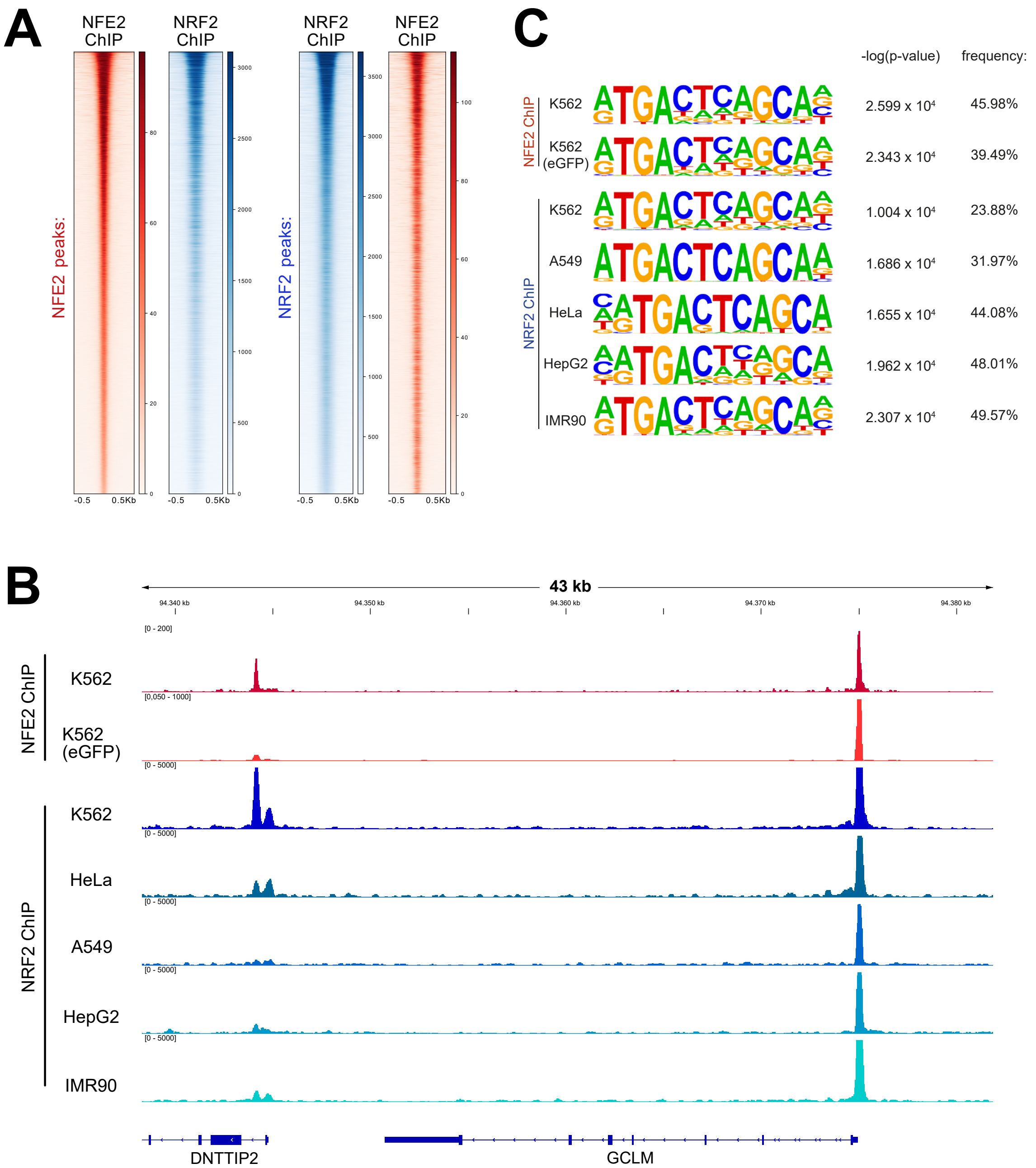

Suppl. Fig. 3: Analysis of NFE2 and NRF2 genome-wide co-occupancy
(**A**) Heatmaps of NFE2 and NRF2 ChIP-seq coverage signal (RPKM) in K562 cells (ENCODE ENCSR000FCC and ENCSR485GKE respectively). 1 kb windows centered around NFE2 (left: n=17313 regions) and NRF2 (right: n=11815 regions) peaks were called at $p < 10^{-10}$ cutoff. **(B)** Representative NFE2 (top) and NRF2 (bottom) ChIP-seq tracks at the *GCLM* locus, coverage scores were normalized as RPKM, data was mapped to hg19. In addition to K562 from (A), ENCODE datasets ENCSR552YGL, ENCSR707IUN, ENCSR584GHV, ENCSR488EES and ENCSR197WGI were used. **(C)** Results of HOMER *de novo* motif analyses in 50 bp windows around NFE2 and NRF2 peak summits at $p < 10^{-5}$. Motif positional weight matrices, significance and frequency shown.

Suppl. Fig. 4

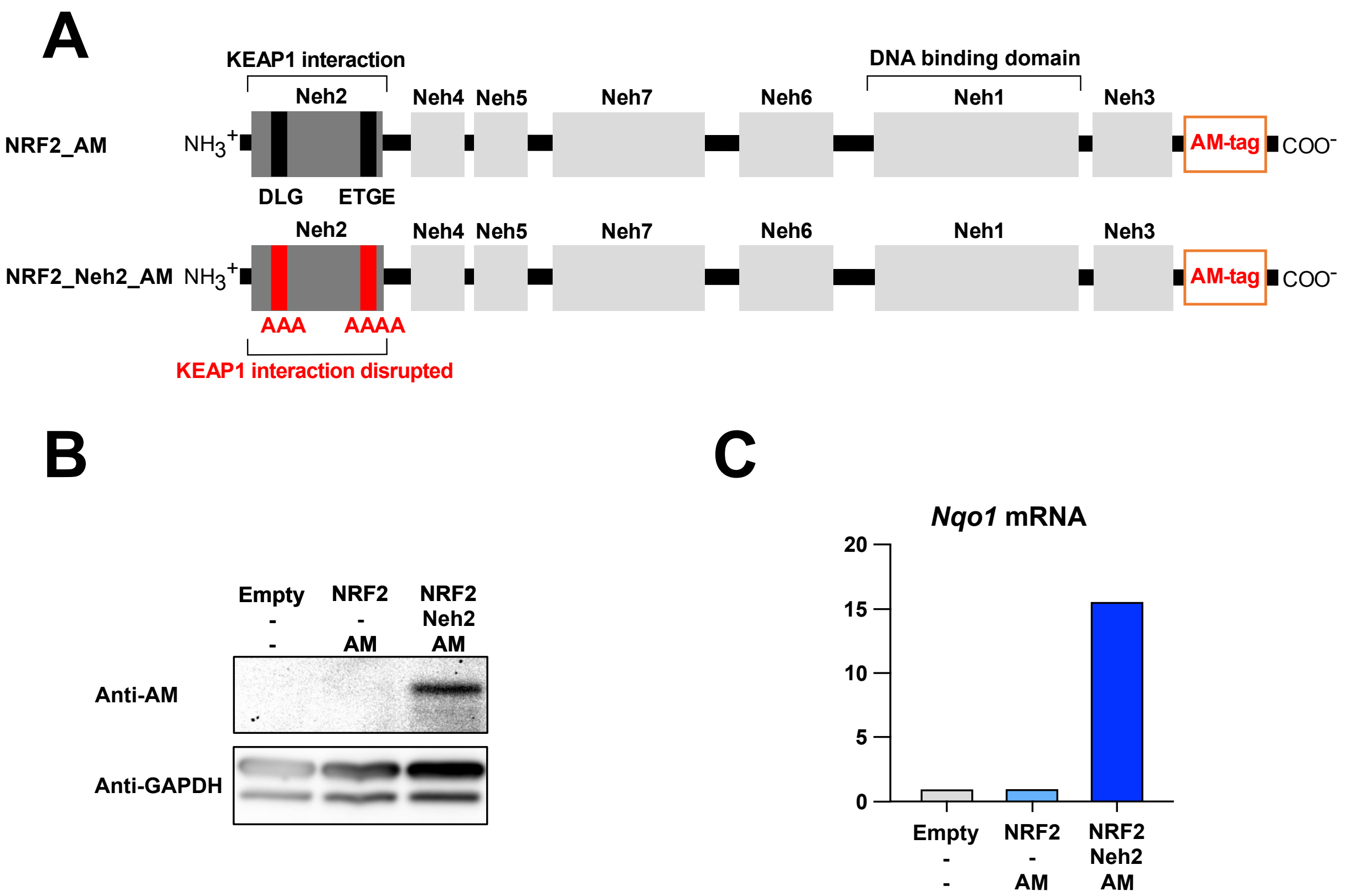

Suppl. Fig. 4: Design and validation of a degradation-resistant AM-tagged NRF2 construct
(**A**) Protein structure of an AM-tagged NRF2 construct (NRF2_AM) and of a degradation resistant Neh2-mutated and AM-tagged NRF2 construct (NRF2_Neh2_AM). The mutation of the Neh2 domain was performed as previously described,[48] mutated amino acids are marked in red**. (B-C)** CB3 cells were lentivirally transduced with an empty vector and vectors overexpressing NRF2_AM or NRF2_Neh2_AM, n = 1 **(B)** Western Blot using Anti-AM and Anti-GAPDH antibodies. **(C)** *Nqo1* mRNA expression measured by RTqPCR. Fold Change is depicted compared to the empty condition.

Suppl. Fig. 5

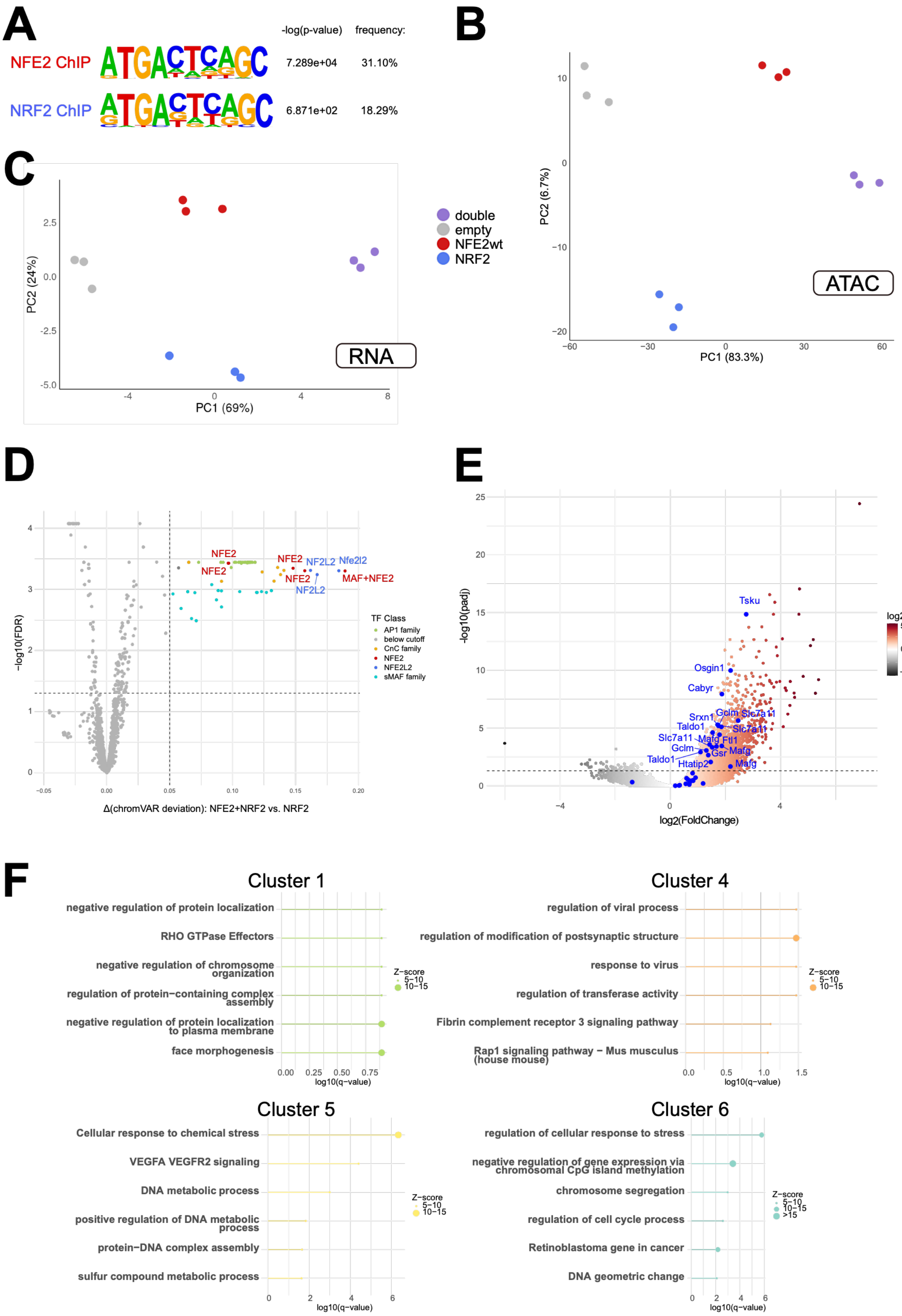

A
-log(p-value)
frequency:
NFE2 ChIP
ATGACTCAGC
7.289e+04
31.10%
NRF2 ChIP
ATGACTCAGC
6.871e+02
18.29%
B
PC2 (6.7%)
PC1 (83.3%)
ATAC
C
PC2 (24%)
PC1 (69%)
RNA
double
empty
NFE2wt
NRF2
D
-log10(FDR)
Δ(chromVAR deviation): NFE2+NRF2 vs. NRF2
NFE2
NF2L2
Nfe2l2
MAF+NFE2
TF Class
AP1 family
below cutoff
CnC family
NFE2
NFE2L2
sMAF family
E
-log10(padj)
log2(FoldChange)
log2FC
Tsku
Osgin1
Cabyr
Srxn1
Gclm
Slc7a11
Taldo1
Mafg
Ftl1
Gsr
Htatip2
F
Cluster 1
negative regulation of protein localization
RHO GTPase Effectors
negative regulation of chromosome organization
regulation of protein−containing complex assembly
negative regulation of protein localization to plasma membrane
face morphogenesis
log10(q-value)
Z-score
5−10
10−15
Cluster 4
regulation of viral process
regulation of modification of postsynaptic structure
response to virus
regulation of transferase activity
Fibrin complement receptor 3 signaling pathway
Rap1 signaling pathway − Mus musculus (house mouse)
log10(q-value)
Cluster 5
Cellular response to chemical stress
VEGFA VEGFR2 signaling
DNA metabolic process
positive regulation of DNA metabolic process
protein−DNA complex assembly
sulfur compound metabolic process
log10(q-value)
Cluster 6
regulation of cellular response to stress
negative regulation of gene expression via chromosomal CpG island methylation
chromosome segregation
regulation of cell cycle process
Retinoblastoma gene in cancer
DNA geometric change
log10(q-value)
>15

Suppl. Fig.5: Additional characterization of simultaneous co-expression of NFE2 and NRF2 in CB3 cells.

(A) Top hits from HOMER *de novo* motif enrichment analysis on NFE2 and NRF2 ChIP-seq (n = 1 each) in CB3 cells are shown. HOMER was run on 50bp windows around NFE2 (GeneTex) and NRF2::AM peaks (anti-AM) peak summits at $p<10^{-5}$. Motif positional weight matrices, significance and frequency shown. **(B)** ATAC-seq: PCA plot of n=500 most variable CREs across conditions (empty, NFE2, NRF2, NFE2 and NRF2 co-expression) by log (CPM) values in CB3 cells, n = 3 biological replicates per condition. **(C)** RNA-seq: PCA plot of n=200 most variable genes across conditions by VST-transformed, normalized counts, n = 3 biological replicates per condition **(D)** ChromVAR TF motif usage analysis in NRF2 and NFE2 co-transduced vs NRF2-overexpressing CB3 cells. Significance was evaluated using two-sided t-test followed by BH-correction. TF families as well as NFE2- and NRF2-specific motifs are highlighted. **(E)** Differential changes in chromatin accessibility between NRF2 and NFE2 co-transduced vs NRF2 overexpressing CB3 cells. Significance was evaluated by Quasi-likelihood F-test and by BH correction for multiple testing. **(F)** Dotplots of gene set enrichment analysis performed on genes in clusters 1, 4, 5 and 6 (Fig. 3E) using Metascape. The six highest-ranking gene sets by significance are displayed, the x-axes display $-\log10(p_{adj})$.

Suppl. Fig. 6

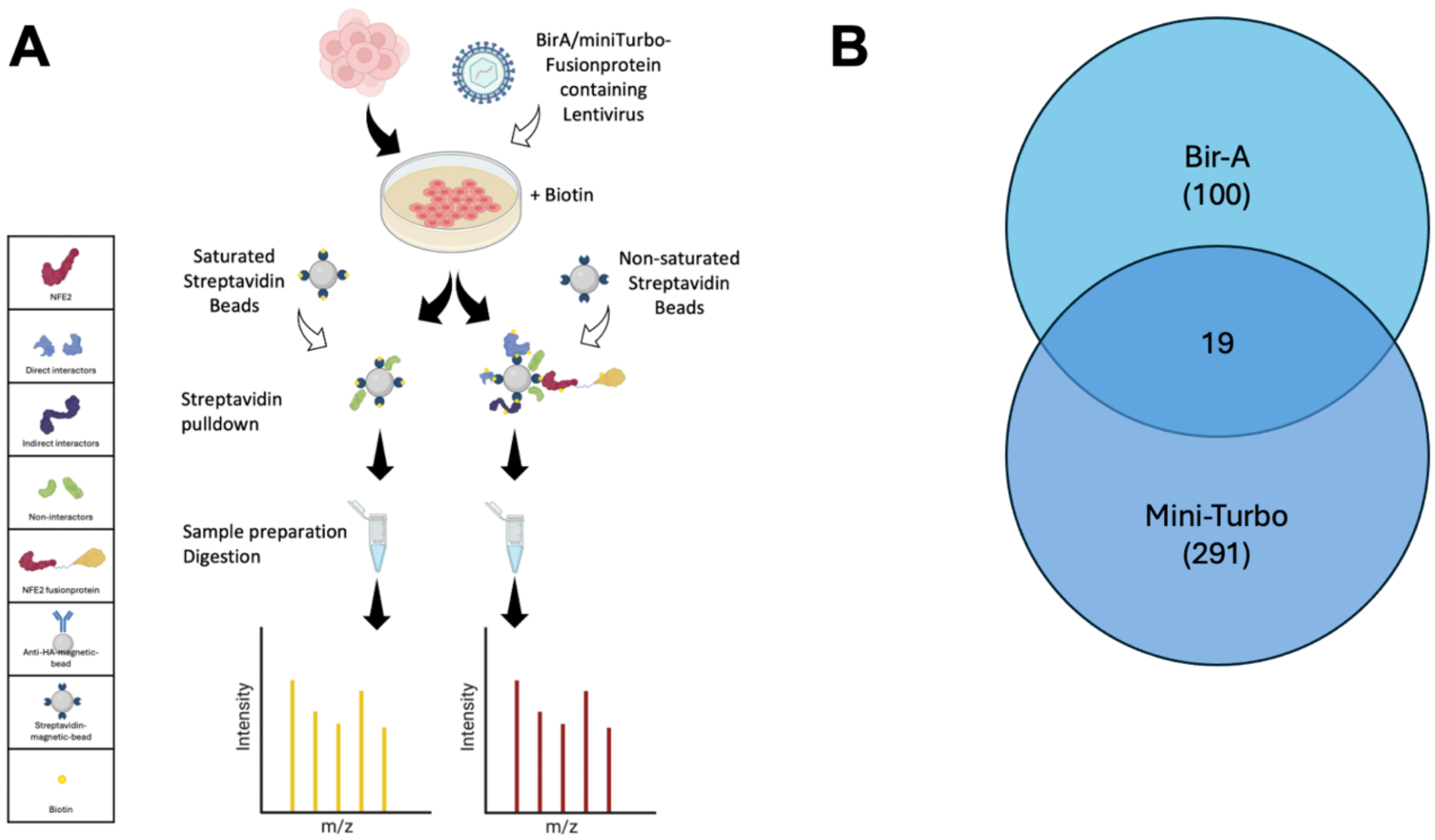

Suppl. Fig. 6: Identification of NFE2 interaction partners using BioID proximity assays
(A) Schematic of the BioID proximity labeling workflow. Cells were transduced with lentiviruses encoding either NFE2-BirA or NFE2-MiniTurbo fusion proteins. Biotinylated proteins were enriched using streptavidin magnetic beads and subsequently identified by mass spectrometry following on-bead tryptic digestion. Created in BioRender. Pahl, H. (2026) *https://BioRender.com/cblk29h* **(B)** Venn diagram summarizing enriched proteins in transduced CB3 cells. Blue circles represent proteins detected with at least 2 unique peptide fragments and a Log2FC of ≥ 0.5 relative to controls (biotin-saturated streptavidin beads). Nineteen shared interacting proteins met these criteria in both NFE2-BirA and NFE2-MiniTurbo pulldowns. Each experimental condition and control included n = 3 independent replicates.

Suppl. Fig. 7

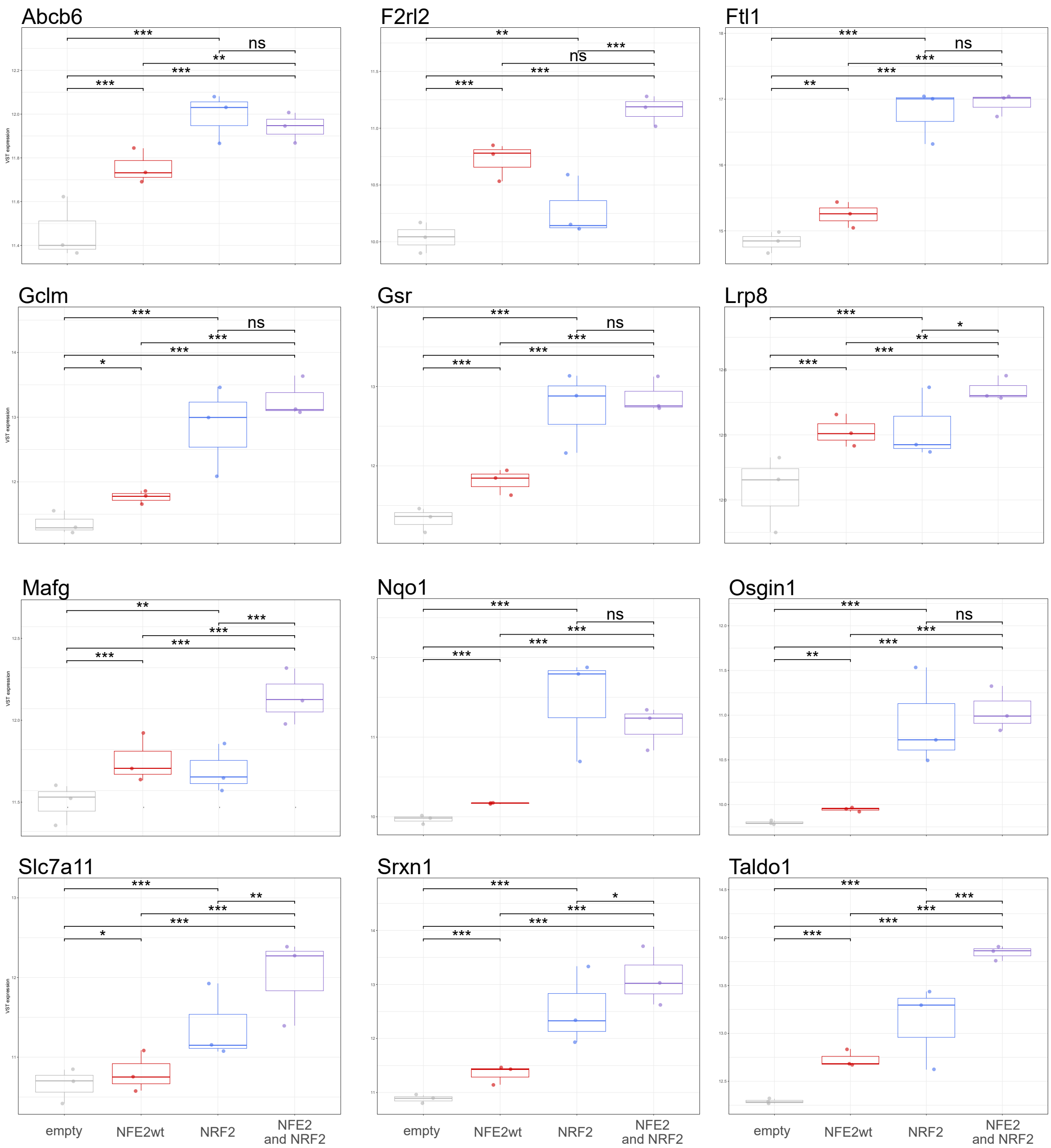

Suppl. Fig. 7: Expression of shared NFE2/NRF2 target genes across different levels of NFE2 and NRF2 overexpression

VST-normalized counts of NRF2 target genes (Conrad *et al.*) in CB3 cells. Genes were subset for those that are differentially upregulated upon NFE2 overexpression (Fig. 1) and whose promoter is NFE2-bound (Fig.2). p_{adj} values for pairwise contrasts are shown above brackets. * $p_{adj} < 0.05$, ** $p_{adj} < 0.01$, *** $p_{adj} < 0.001$, **** $p_{adj} < 0.0001$. Statistical significance was tested via Wald-test (DESeq2). N = 3 biological replicates per condition. Box plot elements: Median, IQRs, max. whisker extension = 1.5 IQR.

Suppl. Fig. 8

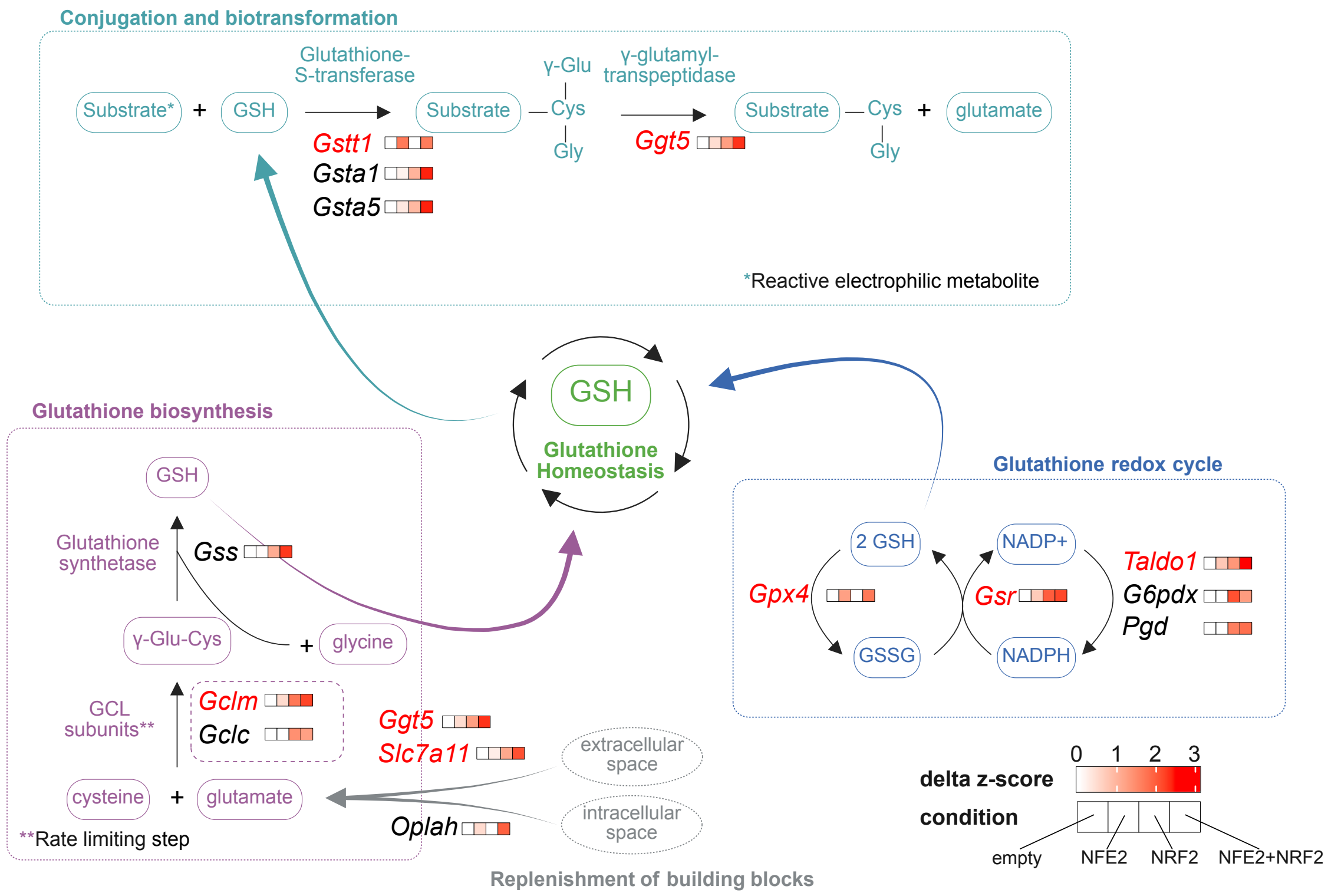

Suppl. Fig. 8: NFE2 overactivity leads to increased expression of central regulators of glutathione homeostasis.
Schematic of reactions involved in glutathione (GSH) homeostasis. For each gene, expression levels from the RNA-seq experiment in Fig. 3 (CB3 + empty/NFE2/NRF2/NFE2+NRF2, n = 3 biological replicates per condition) are depicted as delta z-score of vst-counts. Delta z-scores ≤ 0 are depicted in white. Genes that are significantly upregulated in the NFE2 condition compared to empty are written in red. Created in BioRender. Pahl, H. (2026) *https://BioRender.com/tkbj75f*

Suppl. Fig. 9

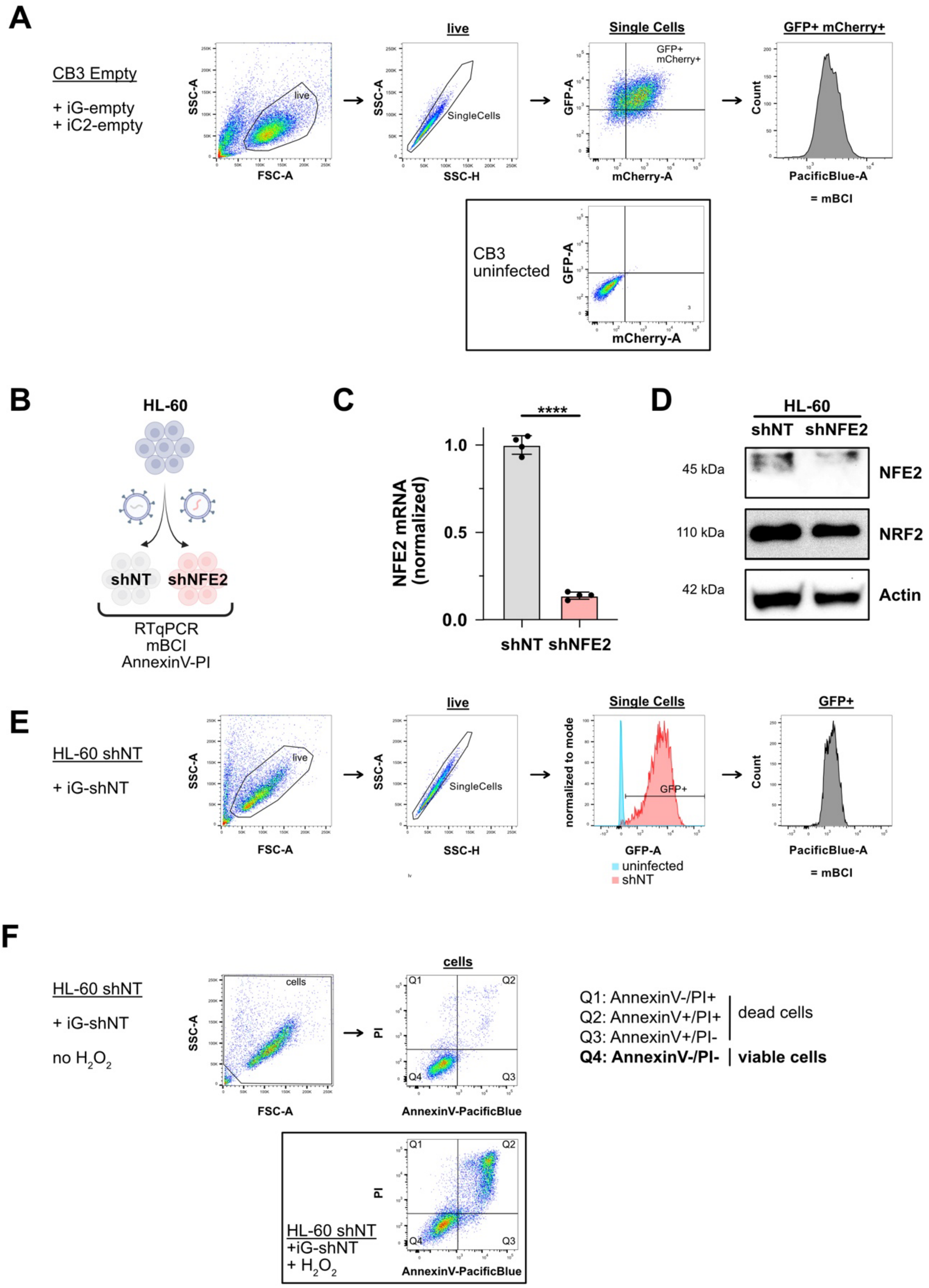

Suppl. Fig. 9: Additional data on glutathione and cell death stainings
(A) Gating strategy for mBCI staining of CB3 cells shown in Fig.4A-B. Uninfected CB3 cells were used to set the GFP+/mCherry+ Gate. mBCI fluorescence was measured in the PacificBlue Channel (Ex

405nm, 450/40 Filter) **(B)** Setup of experiments displayed in Fig.4 C-H. HL-60 cells were transduced with lentiviral vectors expressing a non-targeting shRNA (shNT) or an shRNA targeting NFE2 (shNFE2). Created in BioRender. Pahl, H. (2026) *https://BioRender.com/1kfphw1* **(C)** Knockdown efficiency in HL-60 assessed by RT-qPCR, n = 4 biological replicates per condition **(D)** Western Blot in shNT and shNFE2 expressing HL-60 cells. Primary antibodies against NFE2 (Proteintech), NRF2 (Abcam) and beta actin were used. **(E)** Gating strategy for mBCI staining of HL-60 cells shown in Fig.4C. Uninfected HL-60 cells were used to set the GFP+ gate. **(F)** Gating strategy for AnnexinV-PI staining of HL-60 cells shown in Fig.4G-H. Cell debris was removed by SSC-A/FSC-A gating. Viable cells were defined as AnnexinV-/PI-.

Suppl. Fig. 10

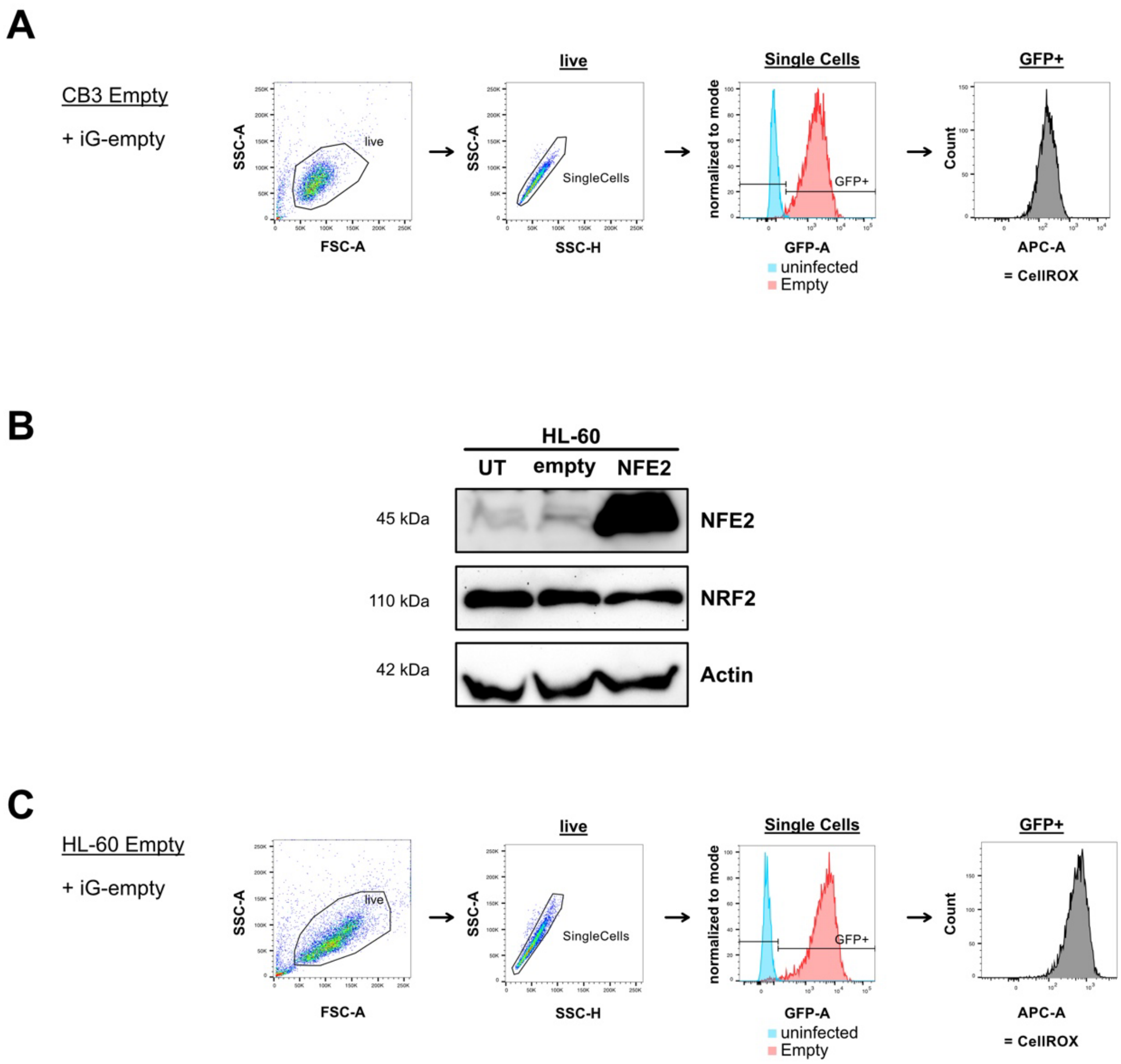

Suppl. Fig. 10. Additional Data on ROS measurements in CB3 and HL-60 cells.
(A) Gating strategy for CellROX staining of CB3 cells shown in Fig.4I-J. Uninfected CB3 cells were used to set the GFP+ Gate. CellROX fluorescence was measured in the APC Channel (Ex 640 nm, 670/14 Filter) **(B)** Western Blot in untransduced (UT) HL-60 cells and HL-60 cells transduced with an empty control vector or with a vector encoding NFE2. Primary antibodies against NFE2 (Proteintech), NRF2 (Abcam) and beta actin were used. **(C)** Gating strategy for CellROX staining of HL-60 cells shown in Fig.4K. Uninfected HL-60 cells were used to set the GFP+ Gate. CellROX fluorescence was measured in the APC Channel (Ex 640 nm, 670/14 Filter)

Suppl. Fig. 11

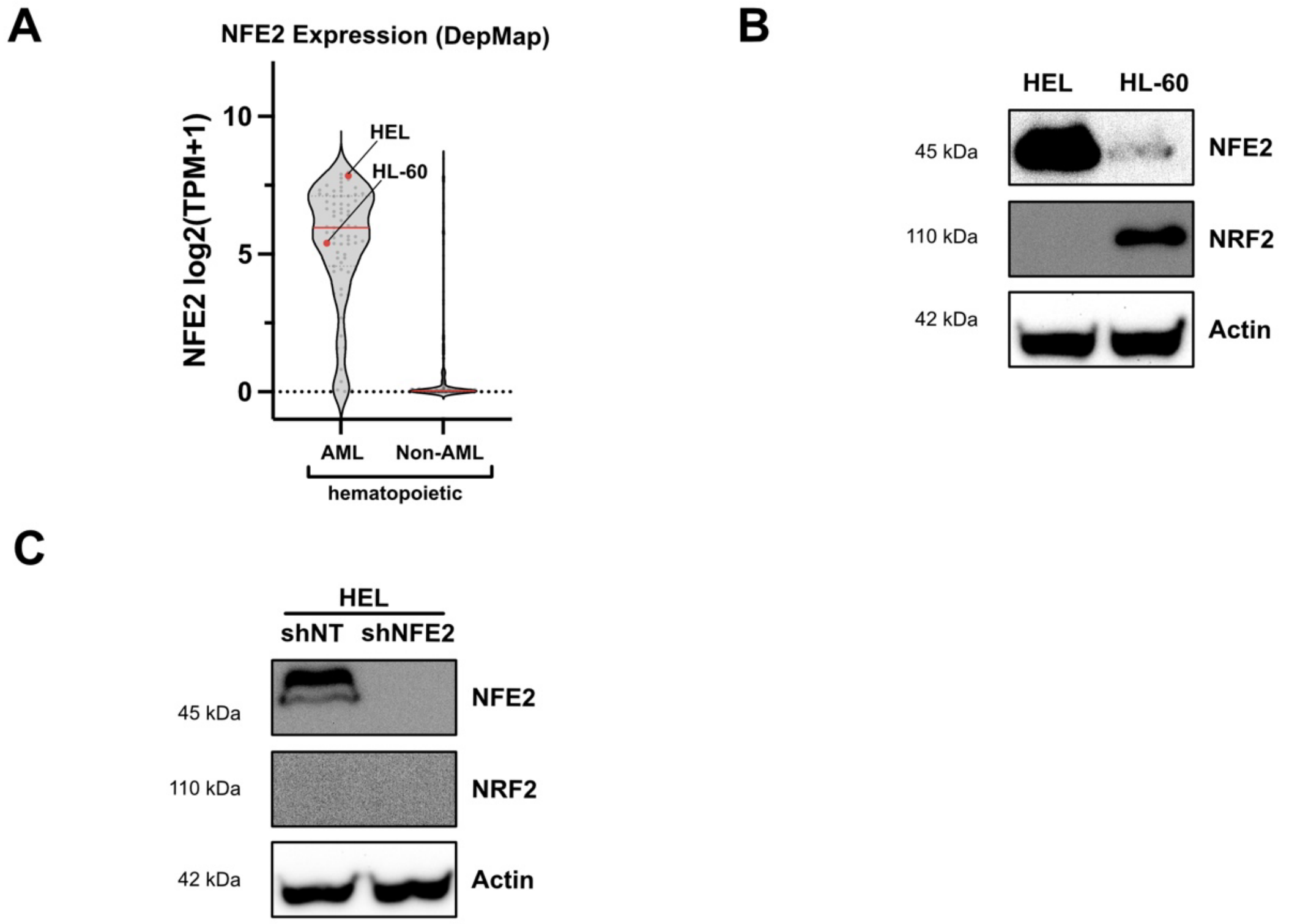

Suppl. Fig. 11: NFE2 and NRF2 expression in HEL and HL-60 cells.
(**A**) NFE2 expression in AML cell lines and non-AML hematopoietic cell lines mining gene expression data from the DepMap portal (DepMap Public 26Q1). AML cell lines (n=64) were selected using the following filter: OncotreePrimaryDisease = Acute Myeloid Leukemia. Non-AML cell lines (n=290) were selected using the following filters: OncotreeLineage = Myeloid or Lymphoid, OncotreePrimaryDisease is not Acute Myeloid Leukemia. HEL and HL-60 are highlighted in red. Median NFE2 expression is depicted as a red line. **(B)** Western Blot of untransduced HEL and HL-60 cells. Primary antibodies against NFE2 (Atlas Antibodies), NRF2 (Abcam) and beta actin were used. **(C)** Western Blot of HEL cells transduced with vectors expressing a non-targeting shRNA (shNT) or an shRNA against NFE2 (shNFE2). Primary antibodies against NFE2 (Atlas Antibodies), NRF2 (Abcam) and beta actin were used.

Suppl. Fig. 12

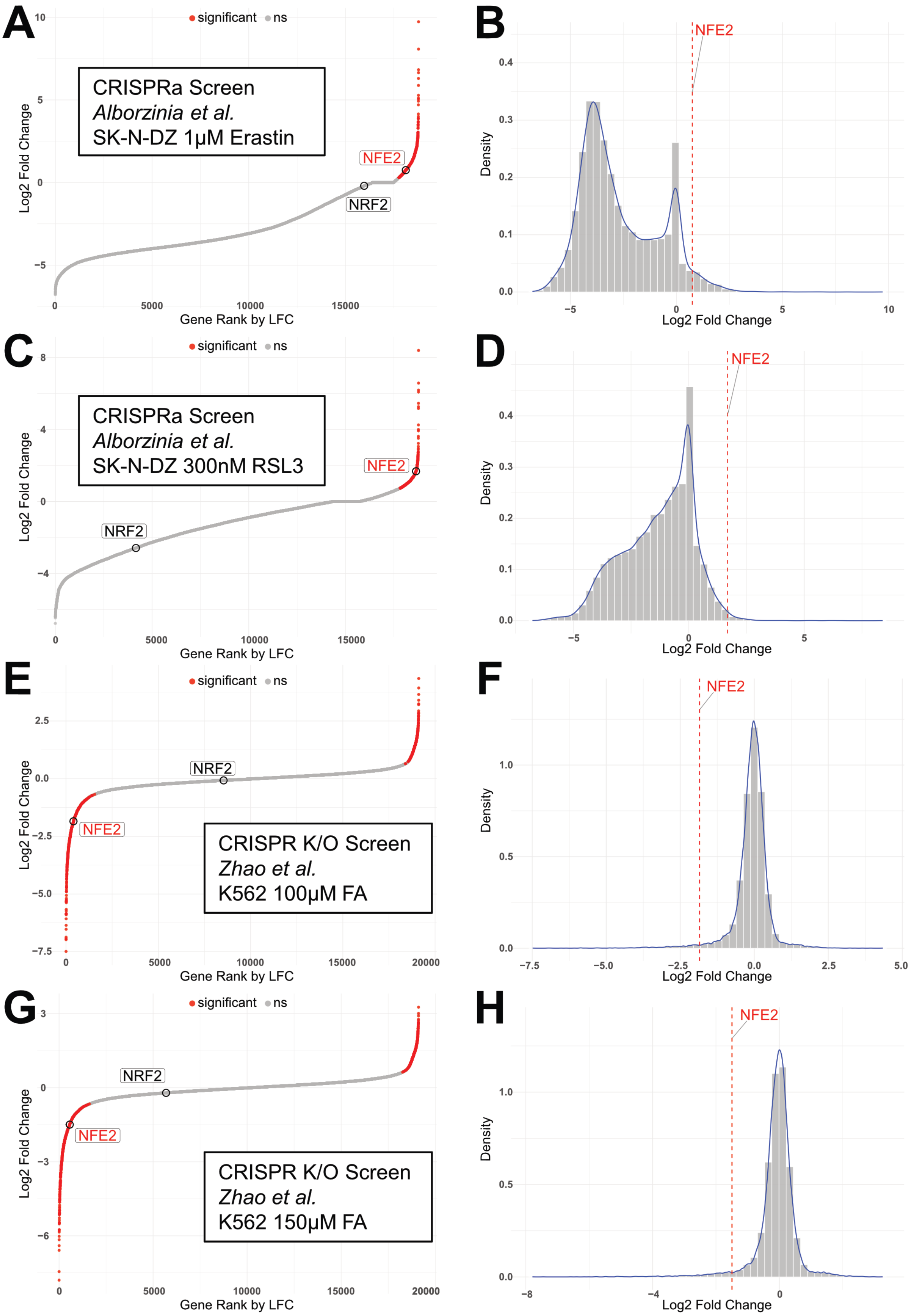
A
B
C
D
E
F
G
H
significant
ns
CRISPRa Screen
Alborzinia et al.
SK-N-DZ 1µM Erastin
CRISPRa Screen
Alborzinia et al.
SK-N-DZ 300nM RSL3
CRISPR K/O Screen
Zhao et al.
K562 100µM FA
CRISPR K/O Screen
Zhao et al.
K562 150µM FA
NFE2
NRF2
Log2 Fold Change
Gene Rank by LFC
Density

Suppl. Fig. 12: NFE2 protects SK-N-DZ cells from ferroptosis induction using Erastin as well as RSL-3 and K562 cells from Formaldehyde-induced redox stress.

(A-D) Publicly available CRISPR activation (CRISPRa) screens of SK-N-DZ cells treated with 1μM Erastin (A-B) or 300nM RSL3 (C-D) for 14 days.[45] Data analyzed using MaGeCK was accessed from BioGRID ORCS. Significant hits are defined as FDR < 0.05. **(E-H)** Publicly available CRISPR knockout (CRISPR K/O) screens of K562 cells treated with 100 μM (E-F) or 150 μM (G-H) Formaldehyde (FA) for 8 days.[46] Data analyzed using EdgeR was accessed from BioGRID ORCS. Significant hits are defined by $|Log2(FC)| > 0.05$ and FDR < 0.01. (**A, C, E, G)** Genes were ranked by Log2 Fold Change. Significant hits are depicted in red. **(B, D, F, H)** Histogram of Log2 Fold Change values (50 bins) with overlaid kernel density estimate. Dashed vertical line marks Log2 Fold Change corresponding to NFE2 (red).

Suppl. Fig. 13

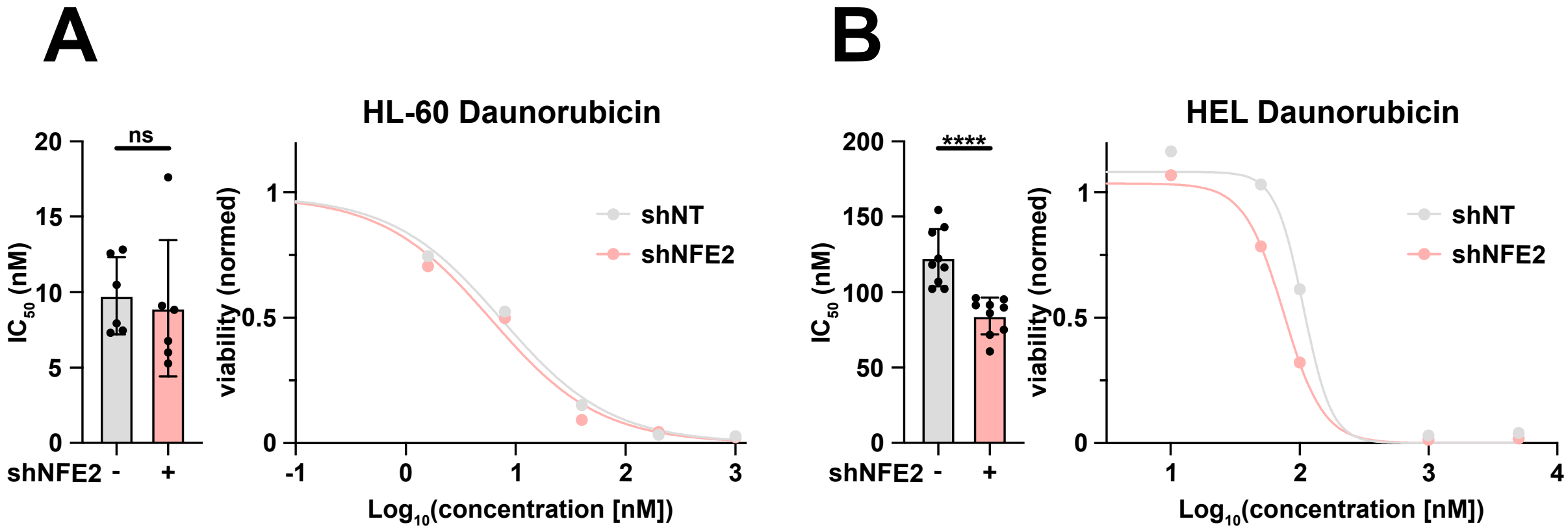

Suppl. Fig. 13: NFE2 depletion sensitizes HEL but not HL-60 to Daunorubicin.
HL-60 (n= 6 biological replicates, A) and HEL (n = 9 biological replicates, B) cells were lentivirally transduced with a non-targeting shRNA (shNT) or an shRNA targeting NFE2 (shNFE2) vector. Daunorubicin treatments were initiated 48h post-transduction. Viability was measured 48 h after Daunorubicin treatment using CellTiter-Glo, normalized to untreated controls, and IC_{50} values were obtained by nonlinear regression in GraphPad Prism 10. Each panel displays the dose–response curve alongside the corresponding IC_{50} bar chart. Representative dose–response curves are shown from one replicate per condition. Statistical analyses were performed using two-sided, unpaired t-tests. * $p < 0.05$, ** $p < 0.01$, *** $p < 0.001$, **** $p < 0.0001$. Mean with SD depicted.

Suppl. Fig. 14

A

Characteristic	Overall N = 446	High N = 223	Low N = 223
**sex**			
Female	202 (45%)	95 (43%)	107 (48%)
Male	244 (55%)	128 (57%)	116 (52%)
**age_quartiles**			
middle	114 (26%)	54 (24%)	60 (27%)
older	103 (23%)	63 (28%)	40 (18%)
oldest	111 (25%)	79 (36%)	32 (14%)
young	116 (26%)	26 (12%)	90 (41%)
Unknown	2	1	1
**eln_2022**			
Adverse	164 (39%)	130 (60%)	34 (17%)
Favorable	115 (27%)	27 (12%)	88 (43%)
Intermediate	143 (34%)	61 (28%)	82 (40%)
Unknown	24	5	19

B

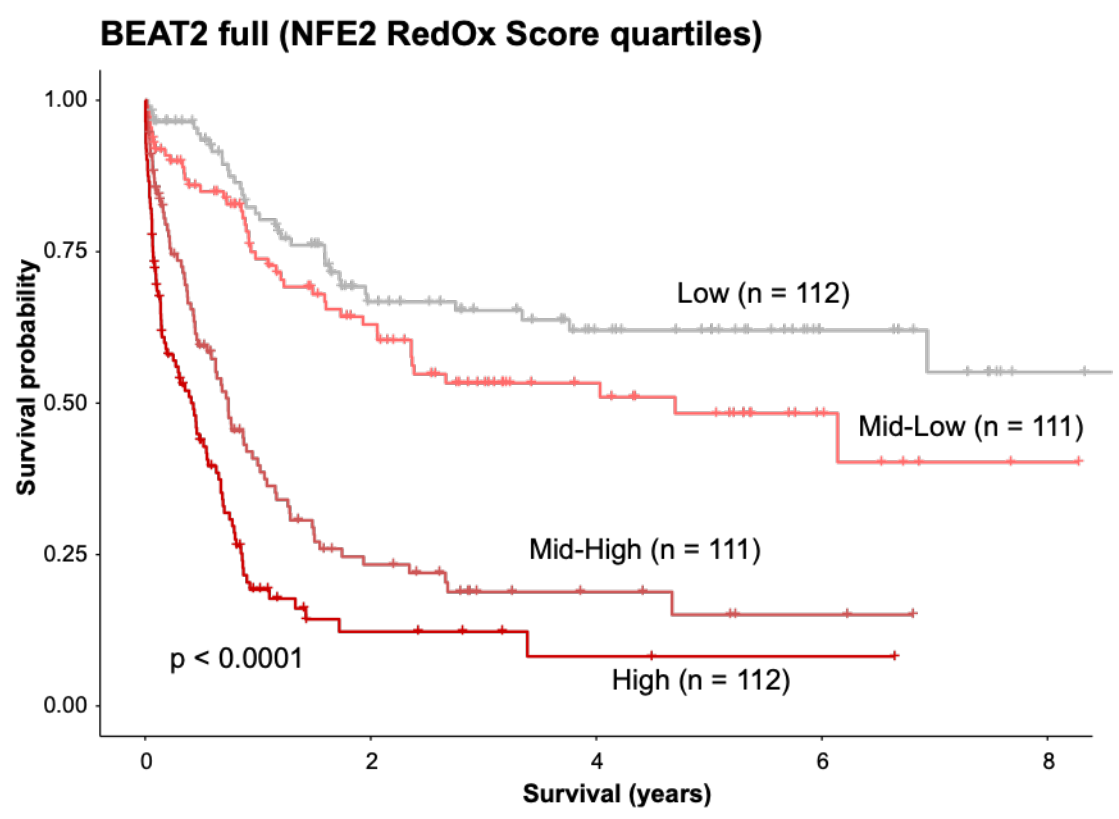

C

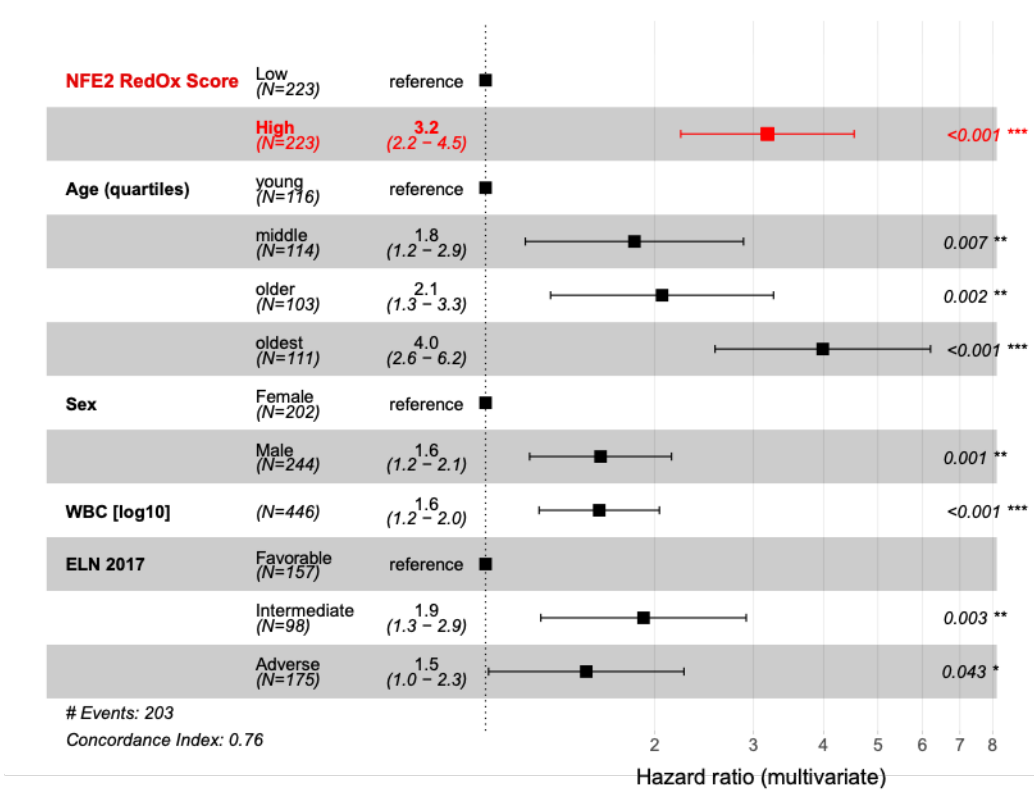

D

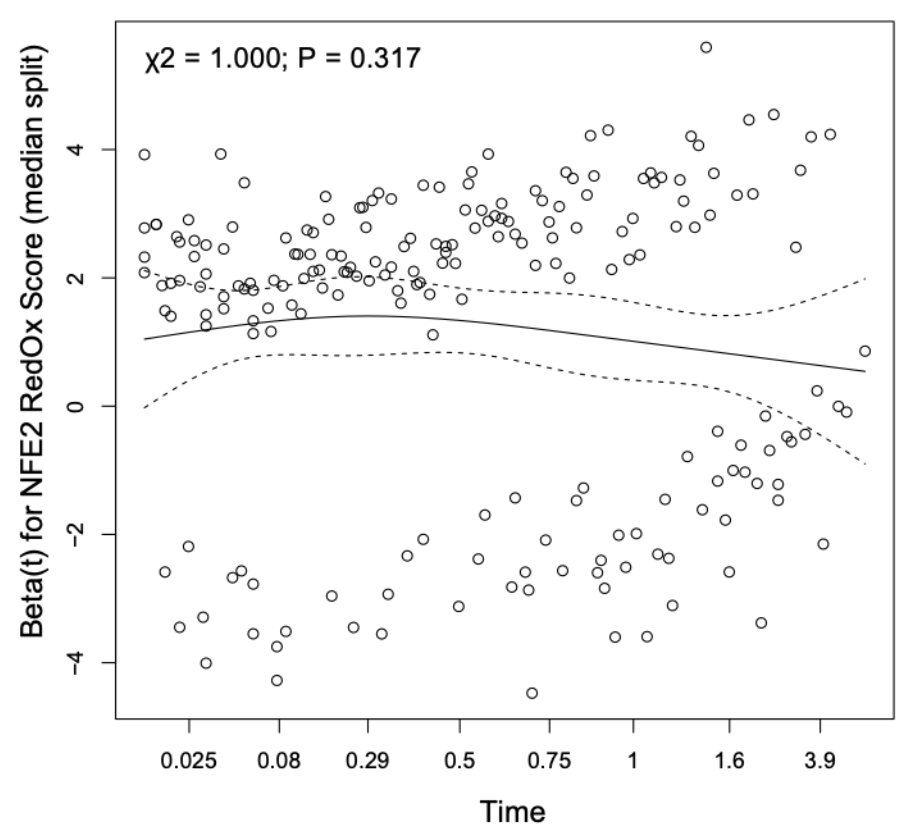

E

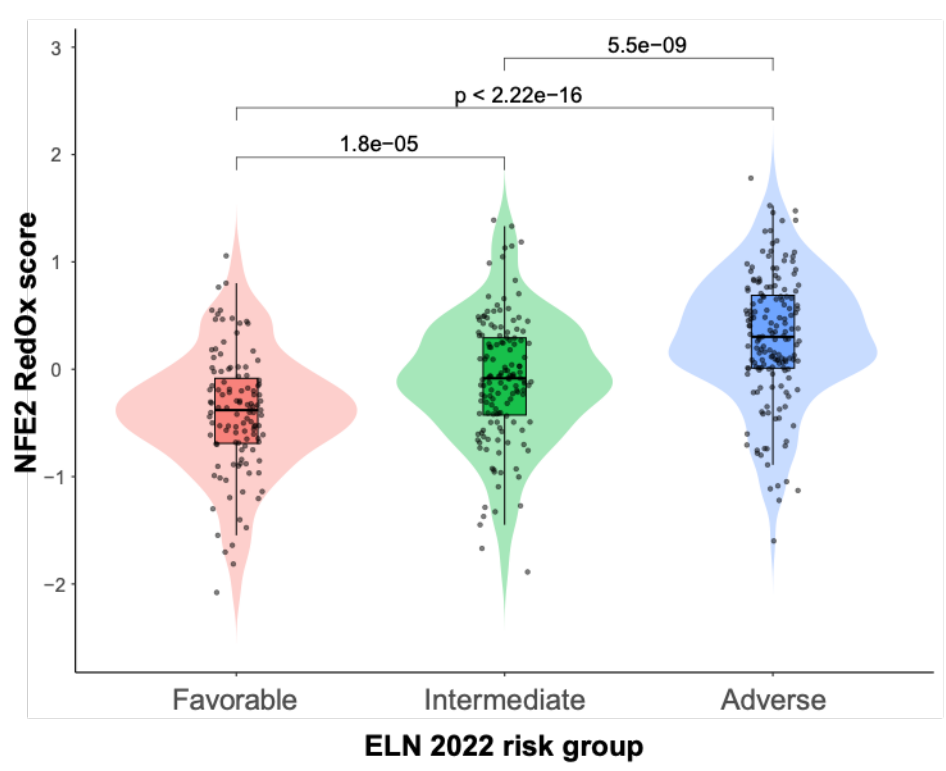

Suppl. Fig. 14: Characterization of the NFE2 RedOx score in the BEAT AML 2 training cohort **(A)** Baseline characteristics of BEAT AML 2 diagnostic samples stratified by high vs. low NFE2 RedOx score (median split), showing distribution across categorical subgroups (age quartiles, sex, ELN 2022 risk group). **(B)** Kaplan-Meier OS in BEAT AML 2 diagnostic samples stratified by NFE2 RedOx score quartile split. **(C)** Forest plot of HRs with 95% CIs from a multivariate CoxPH model in the BEAT AML cohort, including the NFE2 RedOx score (median split) and clinical covariates (age, sex, WBC, ELN 2017 risk category). **(D)** Estimated time-dependent effect of the NFE2 RedOx score in the multivariable

Cox proportional hazards model summarized in Fig. 7F, derived from scaled Schoenfeld residuals. Deviation from proportional hazards for this covariate was assessed using a chi-squared test. **(E)** Box plot showing the NFE2 RedOx score across diagnostic BEAT AML RNA-seq samples stratified by ELN 2022 risk category. Statistical significance was assessed using Wilcoxon rank-sum test.

SUPPLEMENTAL REFERENCES

1 Lu SJ, Rowan S, Bani MR, Ben-David Y. Retroviral integration within the Fli-2 locus results in inactivation of the erythroid transcription factor NF-E2 in Friend erythroleukemias: evidence that NF-E2 is essential for globin expression. *Proc Natl Acad Sci U S A* 1994; **91**: 8398–8402.

2 Wehrle J, Seeger TS, Schwemmers S, Pfeifer D, Bulashevska A, Pahl HL. Transcription factor nuclear factor erythroid-2 mediates expression of the cytokine interleukin 8, a known predictor of inferior outcome in patients with myeloproliferative neoplasms. *Haematologica* 2013; **98**: 1073–1080.

3 Roelz R, Pilz IH, Mutschler M, Pahl HL. Of mice and men: human RNA polymerase III promoter U6 is more efficient than its murine homologue for shRNA expression from a lentiviral vector in both human and murine progenitor cells. *Exp Hematol* 2010; **38**: 792–797.

4 Staehle AM, Peeken JC, Vladimirov G, Hoeness ME, Bojtine Kovacs S, Karantzelis N *et al.* The histone demethylase JMJD2C constitutes a novel NFE2 target gene that is required for the survival of JAK2V617F mutated cells. *Leukemia* 2023; **37**: 919–923.

5 Krivtsov AV, Twomey D, Feng Z, Stubbs MC, Wang Y, Faber J *et al.* Transformation from committed progenitor to leukaemia stem cell initiated by MLL-AF9. *Nature* 2006; **442**: 818–822.

6 Aubrey BJ, Cutler JA, Bourgeois W, Donovan KA, Gu S, Hatton C *et al.* IKAROS and MENIN coordinate therapeutically actionable leukemogenic gene expression in MLL-r acute myeloid leukemia. *Nat Cancer* 2022; **3**: 595–613.

7 Gu SS, Zhang W, Wang X, Jiang P, Traugh N, Li Z *et al.* Therapeutically Increasing MHC-I Expression Potentiates Immune Checkpoint Blockade. *Cancer Discov* 2021; **11**: 1524–1541.

8 Perner F, Schnoeder TM, Xiong Y, Jayavelu AK, Mashamba N, Santamaria NT *et al.* YBX1 mediates translation of oncogenic transcripts to control cell competition in AML. *Leukemia* 2022; **36**: 426–437.

9 Chen C-H, Xiao T, Xu H, Jiang P, Meyer CA, Li W *et al.* Improved design and analysis of CRISPR knockout screens. *Bioinformatics* 2018; **34**: 4095–4101.

10 Mechold U, Gilbert C, Ogryzko V. Codon optimization of the BirA enzyme gene leads to higher expression and an improved efficiency of biotinylation of target proteins in mammalian cells. *J Biotechnol* 2005; **116**: 245–249.

11 Branon TC, Bosch JA, Sanchez AD, Udeshi ND, Svinkina T, Carr SA *et al.* Efficient proximity labeling in living cells and organisms with TurboID. *Nat Biotechnol* 2018; **36**: 880–887.

12 Weber K, Bartsch U, Stocking C, Fehse B. A multicolor panel of novel lentiviral 'gene ontology' (LeGO) vectors for functional gene analysis. *Mol Ther* 2008; **16**: 698–706.

13 Spruijt CG, Baymaz HI, Vermeulen M. Identifying specific protein-DNA interactions using SILAC-based quantitative proteomics. *Methods Mol Biol* 2013; **977**: 137–157.

14 Tyanova S, Temu T, Cox J. The MaxQuant computational platform for mass spectrometry-based shotgun proteomics. *Nat Protoc* 2016; **11**: 2301–2319.

15 Schwanhäusser B, Busse D, Li N, Dittmar G, Schuchhardt J, Wolf J *et al.* Global quantification of mammalian gene expression control. *Nature* 2011; **473**: 337–342.

16 Jutzi JS, Basu T, Pellmann M, Kaiser S, Steinemann D, Sanders MA *et al.* Altered NFE2 activity predisposes to leukemic transformation and myelosarcoma with AML-specific aberrations. *Blood* 2019; **133**: 1766–1777.

17 Arrigoni L, Richter AS, Betancourt E, Bruder K, Diehl S, Manke T *et al.* Standardizing chromatin research: a simple and universal method for ChIP-seq. *Nucleic Acids Res* 2016; **44**: e67.

18 Corces MR, Trevino AE, Hamilton EG, Greenside PG, Sinnott-Armstrong NA, Vesuna S *et al.* An improved ATAC-seq protocol reduces background and enables interrogation of frozen tissues. *Nat Methods* 2017; **14**: 959–962.

19 Patro R, Duggal G, Love MI, Irizarry RA, Kingsford C. Salmon provides fast and bias-aware quantification of transcript expression. *Nat Methods* 2017; **14**: 417–419.

20 Soneson C, Love MI, Robinson MD. Differential analyses for RNA-seq: transcript-level estimates improve gene-level inferences. *F1000Res* 2015; **4**: 1521.

21 Love MI, Huber W, Anders S. Moderated estimation of fold change and dispersion for RNA-seq data with DESeq2. *Genome Biol* 2014; **15**: 550.

22 Yu G, Wang L-G, Han Y, He Q-Y. clusterProfiler: an R package for comparing biological themes among gene clusters. *OMICS* 2012; **16**: 284–287.

23 Zhou Y, Zhou B, Pache L, Chang M, Khodabakhshi AH, Tanaseichuk O *et al.* Metascape provides a biologist-oriented resource for the analysis of systems-level datasets. *Nat Commun* 2019; **10**: 1523.

24 Staehle HF, Koellerer C, Staehle AM, Schulze J, Eble P, Müller A *et al.* Lysine-specific demethylase 1 regulates hematopoietic stem cell expansion and myeloid cell differentiation. *Cell Death Dis* 2025; **16**: 619.

25 Langmead B, Salzberg SL. Fast gapped-read alignment with Bowtie 2. *Nat Methods* 2012; **9**: 357–359.

26 Tarasov A, Vilella AJ, Cuppen E, Nijman IJ, Prins P. Sambamba: fast processing of NGS alignment formats. *Bioinformatics* 2015; **31**: 2032–2034.

27 Ramírez F, Ryan DP, Grüning B, Bhardwaj V, Kilpert F, Richter AS *et al.* deepTools2: a next generation web server for deep-sequencing data analysis. *Nucleic Acids Res* 2016; **44**: W160-165.

28 Feng J, Liu T, Qin B, Zhang Y, Liu XS. Identifying ChIP-seq enrichment using MACS. *Nat Protoc* 2012; **7**: 1728–1740.

29 Amemiya HM, Kundaje A, Boyle AP. The ENCODE Blacklist: Identification of Problematic Regions of the Genome. *Sci Rep* 2019; **9**: 9354.

30 Zerbino DR, Johnson N, Juettemann T, Wilder SP, Flicek P. WiggleTools: parallel processing of large collections of genome-wide datasets for visualization and statistical analysis. *Bioinformatics* 2014; **30**: 1008–1009.

31 Lun ATL, Smyth GK. csaw: a Bioconductor package for differential binding analysis of ChIP-seq data using sliding windows. *Nucleic Acids Res* 2016; **44**: e45.

32 Schep AN, Wu B, Buenrostro JD, Greenleaf WJ. chromVAR: inferring transcription-factor-associated accessibility from single-cell epigenomic data. *Nat Methods* 2017; **14**: 975–978.

33 Rauluseviciute I, Riudavets-Puig R, Blanc-Mathieu R, Castro-Mondragon JA, Ferenc K, Kumar V *et al.* JASPAR 2024: 20th anniversary of the open-access database of transcription factor binding profiles. *Nucleic Acids Res* 2024; **52**: D174–D182.

34 Corces MR, Granja JM, Shams S, Louie BH, Seoane JA, Zhou W *et al.* The chromatin accessibility landscape of primary human cancers. *Science* 2018; **362**: eaav1898.

35 Lawrence M, Huber W, Pagès H, Aboyoun P, Carlson M, Gentleman R *et al.* Software for computing and annotating genomic ranges. *PLoS Comput Biol* 2013; **9**: e1003118.

36 Bottomly D, Long N, Schultz AR, Kurtz SE, Tognon CE, Johnson K *et al.* Integrative analysis of drug response and clinical outcome in acute myeloid leukemia. *Cancer Cell* 2022; **40**: 850-864.e9.

37 Holmström S, Hautaniemi S, Häkkinen A. POIBM: batch correction of heterogeneous RNA-seq datasets through latent sample matching. *Bioinformatics* 2022; **38**: 2474–2480.

38 Tay JK, Narasimhan B, Hastie T. Elastic Net Regularization Paths for All Generalized Linear Models. *J Stat Softw* 2023; **106**: 1.

39 Cancer Genome Atlas Research Network, Ley TJ, Miller C, Ding L, Raphael BJ, Mungall AJ *et al.* Genomic and epigenomic landscapes of adult de novo acute myeloid leukemia. *N Engl J Med* 2013; **368**: 2059–2074.

40 Verhaak RGW, Wouters BJ, Erpelinck CAJ, Abbas S, Beverloo HB, Lugthart S *et al.* Prediction of molecular subtypes in acute myeloid leukemia based on gene expression profiling. *Haematologica* 2009; **94**: 131–134.

41 Bolouri H, Farrar JE, Triche T, Ries RE, Lim EL, Alonzo TA *et al.* The molecular landscape of pediatric acute myeloid leukemia reveals recurrent structural alterations and age-specific mutational interactions. *Nat Med* 2018; **24**: 103–112.

42 Jayavelu AK, Wolf S, Buettner F, Alexe G, Häupl B, Comoglio F *et al.* The proteogenomic subtypes of acute myeloid leukemia. *Cancer Cell* 2022; **40**: 301-317.e12.

43 Kobayashi EH, Suzuki T, Funayama R, Nagashima T, Hayashi M, Sekine H *et al.* Nrf2 suppresses macrophage inflammatory response by blocking proinflammatory cytokine transcription. *Nat Commun* 2016; **7**: 11624.

44 Fu J, Xiong Z, Huang C, Li J, Yang W, Han Y *et al.* Hyperactivity of the transcription factor Nrf2 causes metabolic reprogramming in mouse esophagus. *J Biol Chem* 2019; **294**: 327–340.

45 Alborzinia H, Chen Z, Yildiz U, Freitas FP, Vogel FCE, Varga JP *et al.* LRP8-mediated selenocysteine uptake is a targetable vulnerability in MYCN-amplified neuroblastoma. *EMBO Mol Med* 2023; **15**: e18014.

46 Zhao Y, Wei L, Tagmount A, Loguinov A, Sobh A, Hubbard A *et al.* Applying genome-wide CRISPR to identify known and novel genes and pathways that modulate formaldehyde toxicity. *Chemosphere* 2021; **269**: 128701.

47 Arafeh R, Shibue T, Dempster JM, Hahn WC, Vazquez F. The present and future of the Cancer Dependency Map. *Nat Rev Cancer* 2025; **25**: 59–73.

48 Liu P, Kerins MJ, Tian W, Neupane D, Zhang DD, Ooi A. Differential and overlapping targets of the transcriptional regulators NRF1, NRF2, and NRF3 in human cells. *J Biol Chem* 2019; **294**: 18131–18149.
